# Description of a new species of *Acrostichus* Rahm 1928 (Nematoda: Diplogastridae) from India with a note on its position and relationship with the congeners

**DOI:** 10.3897/BDJ.4.e8029

**Published:** 2016-04-07

**Authors:** Qudsia Tahseen, Shikha Ahlawat, Mohammad Asif, Malka Mustaqim

**Affiliations:** ‡ Department of Zoology, Aligarh Muslim University, Aligarh, India

**Keywords:** *Acrostichus medius* n. sp., description, taxonomy, cladistic relationship

## Abstract

**Background:**

The clade Diplogastridae Micoletzky 1922 largely represents the bacterivorous or predatory nematodes that very often demonstrate phoretic, necromenic or parasitic associations with insects (Sudhaus and Fürst von Lieven 2003). That is the reason, much of the diversity of the family remains undocumented because of their absence from routine soil samples. Due to their variable habitats and niches, these nematodes show ample variation in their stomal armature, feeding behavior and life cycle patterns.

**New information:**

The paper describes and illustrates a new diplogastrid species of genus *Acrostichus* Rahm 1928 that appears to be the link between the genera *Diplogastrellus* Paramonov et al. 1952a and *Acrostichus*. *Acrostichus medius* n. sp. is characterised by hermaphroditic females and males having faintly striated longitudinal ridges, demarcated lateral fields, amalgamated lips, six adradial cheilostomal plates, moderately-built dorsal tooth, relatively smaller posterior genital branch; large oval uterine pouch and males with robust, heavily cuticularised spicules, each with hood-like capitulum, deeply forked distal end with fine extensions and a ventral attenuated arm; gubernaculum 2/3 of spicule length and rudiments of bursa confined to posterior four pairs of genital papillae. The biogeographical distribution of *Acrostichus* has been mapped and the relationship between congeners has been analysed cladistically and discussed.

## Introduction

### About the genus *Acrostichus* Rahm, 1928

The genus *Acrostichus* was raised by Rahm (1928) when he reported and described *Acrostichus toledoi* as its type species. Goodey (1951) synonymised it with the genus *Diplogaster* Schultze in Carus 1857. Later, Paramonov et al. (1952a) raised the genus *Diplogasteritus* (type species *Diplogaster nudicapitatus* Steiner 1914) based on the presence of longitudinal cuticular ridges, narrow and long stoma, distinctly separated cheilostom from rest of stoma, presence of a dorsal tooth distinctly larger than subventral teeth. *Diplogasteritus* was demarcated by another genus *Diplogastrellus* in the number of genital branches which were paired (opposed) in the former and single (anterior) in the latter. Paramonov et al. 1952a inсludеd ninе spесiеs in *Diplogаstеritus viz*., *D. nudiсаpitatus* (Steiner 1914), *D. filiсаudаtus* (Bütschli 1874), *D. rhodani* (Steiner 1914), *D. consobrinus* (De Man 1920), *D. demаni* (Schneider 1923), *D. minutus* (Kreis 1929), *D. аustriасus* (Fuchs 1938), *D. supеrbus* (Paesler 1946) and *D. oсcidentalis* (Steiner 1932). However, in his monograph, Paramonоv (1964) inсludеd fifteen valid spесiеs in *Diplogаstеritus*. Weingärtner (1950) considered eight species *viz.*, *Diplоgаstеr (Diplogastеr) dепdrophilus* Weingärtner 1950, *D. (D.) сonsobrinus* De Man 1920, *D. (D.) austriасus* Fuchs 1938, *D. (D.) nudiсаpitаtus* Steiner 1914, *D. (D.) supеrbus* Paesler 1946, *D. (D.) stoесkhеrti* Völk 1950, *D. (D.) linеаtus* Fuchs 1915 аnd *D. (D.) subtеrrаnеus* Hnatewytsch 1929 under ‘*nudicapitatus*’ species group under the genus and subgenus *Diplogaster*. Massey (1962) synonymised *Diplogasteritus* with *Acrostichus* considering the former as a junior synonym and recognized a total of nineteen species under it with *A. toledoi* as type species. Goodey (1963) considered *Diplogasteritus* as a valid genus and endorsed Weingärtner (1950) concept of ‘*nudicapitatus*’ group along with listing eight species *viz.*, *D. апgustilаimus* (Schuurmans Stekhoven and Teunissen 1938), *D. brеviсаudаtus* (Schuurmans Stekhoven and Teunissen 1938), *D. еurуcеphаIus* (Völk 1950), *D. lаbiаtus* (Соbb in Merrill and Ford 1916), *D. lirаtus* (Schneider 1866), *D. minutus* (Kreis 1930), *D. occidеntalis* (Steiner 1932) and *D. zurstrаssеni* (Saсhs 1950) thus making the number of species in the genus sixteen, however, he regarded *Acrostichus* as a *genus inquirendum*. Lazarevskaja (1965) splitted *Acrostichus* into *Acrostichus* and *Filipjevella* by placing 5 species under the latter and *F. minimus* Lazarevskaja 1965 as its type species, which was later proposed as a junior synonym of *Diplogasteritus* as well as *Acrostichus*. Andrássy (1984) recognized the two genera, and differentiated *Acrostichus* by having unpaired, anterior female gonad and *Diplogasteritus* by paired female gonad. While considering *Filipjevella* a junior synonym of *Diplogasteritus*, he diagnosed the latter genus with the presence of a large rounded, offset uterine pouch opposite vulva. Andrássy (1984) inсludеd 23 spесiеs in *Diplogаstеritus*. Gagarin (2002) while endorsing Andrássy's view, published a review of the genus *Diplogasteritus* and after several synonymizations considered eight valid species in the genus. Earlier, Fürst von Lieven and Sudhaus (2000) accepted Diplogastrina as a suborder of Rhabditida and gave an outline phylogeny of this taxon based on comparative functional morphology of the buccal cavity. Subsequently, in their catalogue of the Diplogastridae, Sudhaus and Fürst von Lieven (2003) regarded *Diplogasteritus*, *Filipjevella* and *Aduncospiculum* Giblin and Kaya 1984 as junior synonyms of *Acrostichus* and considered 28 valid species under the latter. They also accepted only 28 genera out of the 83 published genera under Diplogastridae. Three more species *A. rhyncophori*, *A. megaloptae* and *A. puri* have been added later to the genus by Kanzaki et al. (2009); Kanzaki et al. (2010b), Kanzaki et al. (2010a) respectively, thus making a total of 31 valid species under *Acrostichus*. Seinhorst et al. (1959b) raised a new family Titilleinae for the new genus *Titilleus* based mainly on structure of gubernaculum and considered *Acrostichus*, *Diplogasteritus*, *Peterngus* Ahmad et al. 2004 and *Aduncospiculum* as other subordinate genera. He also described three new species *viz.*, *D. major*, *D. microsus* and *D. teloporosus* under the genus *Diplogasteritus*.

## Materials and methods

### Collection, processing, extraction and taxonomic study

The soil and water samples were processed using Cobb (1918) sieving and decantation and modified Baermann’s funnel techniques. The nematodes were extracted and fixed in hot formalin-glycerol fixative, dehydrated by the slow evaporation method (Seinhorst et al. 1959a) and mounted in anhydrous glycerine. Permanent slides were prepared using the paraffin wax-ring method (De Maeseneer and D’ Herde 1963). The measurements were taken with an ocular micrometer. LM photographs were taken with a Jenoptik ProgRes digital camera mounted on an Olympus BX-51 DIC microscope. For Scanning Electron Microscopy (SEM), the specimens were fixed in 2% glutaraldehyde, post-fixed in 2% osmium tetroxide, dehydrated in alcohol series and critical point dried using CO_2_. The mounted nematodes were coated with 10 nm gold before viewing at 10 kV with an XL30 FEG scanning electron microscope.

**Figure 1.**
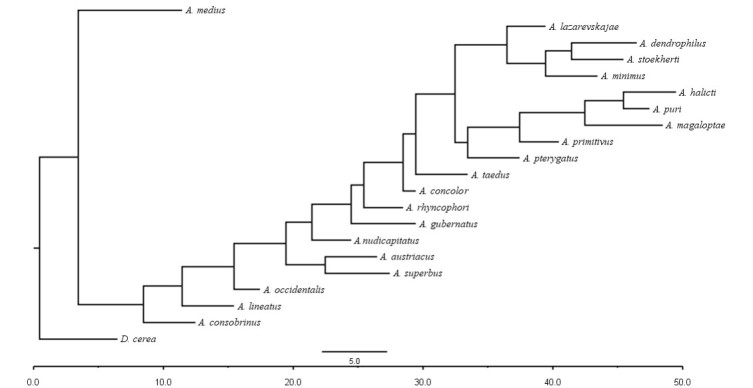
Phylogram showing relationship between species of *Acrostichus* Rahm 1928 based on morphological data with *Diplogastrellus cerea* as an outgroup.

Thirty morphological characters were selected to compare 20 species of *Acrostichus* which showed relatively detailed description (Table 2). The species with insufficient morphological details could not be included in comparison. All the selected characters were important with good taxonomic value. The characters were ranked on the basis of commonality principle. Character state ‘0’’ represented the most commonly occurring trait whereas a gradual increase in value represented more deviation. Data matrix (Table 3) was prepared and single parsimonious tree was retrieved (Fig. 1) for analyses using PAUP (version 4.0 b10) (Swofford 2001) under a parsimony criterion with a heuristic search with TBR (Tree Bisection Reconnection) branch-swapping options.

## Taxon treatments

### Acrostichus medius sp. n.

urn:lsid:zoobank.org:author:E2679573-3F6B-4221-8817-AFB63266528D

#### Materials

- **Type status:** Holotype. **Record Level:** type: Acrostichus medius; modified: NOQ; language: English; rightsHolder: Aligarh Muslim University; bibliographicCitation: Tahseen et al.; institutionID: amu.ac.in; collectionID: urn:lsid:zoobank.org:author:E2679573-3F6B-4221-8817-AFB63266528D; institutionCode: AMU; collectionCode: NOQ

#### Description

Hermaphrodite female (Figs 2, 3, 4, 5, 6, 7 and Table 1): Body slender, medium to large-sized; almost straight after fixation, tapering at both extremities. Cuticle with fine transverse striations; longitudinal ridges delicate, striated (Figs 3e, 4a, e, 5b). Punctations faint, dot-like, running along longitudinal ridges (Fig. 4a). Lateral fields demarcated with two conspicuous ridges about 3–5 µm apart. Lip region continuous with adjoining body. Lips amalgamated; labial sensilla slightly raised. Amphidial apertures elliptical, 2.5–3.5 µm wide and situated about 5–7 µm from anterior end of stoma (Fig. 2c). Cheilostom cuticularised, converging anteriorly with six adradial plates, gymnostom anisomorphic with dorsal wall thickened than sub-ventrals. Stegostom anisotopic, anisomorphic. Dorsal metastegostomal wall with a triangular tooth, each sub-ventral wall provided with relatively smaller tooth (Figs 2c, 3a, b). Pharyngeal corpus muscular, swollen 70–88 µm long; metacorpus, rounded to ovoid strongly developed with thickened lumen, more or less oblong in few specimens; isthmus conspicuously differentiated from metacorpus, 33–45 µm long. Basal bulb small, pyriform, glandular in nature, continuous with isthmus, 16–22 µm x 13–17 µm in dimension (Figs 2d, 3d). Nerve ring encircling isthmus at 67–74% of pharyngeal length from anterior end. Secretory-excretory pore placed posterior to nerve ring or at 81–85% of pharyngeal length from anterior end (Figs 2d, 3e). Body at pharyngeal end 3.5–4.4 times labial diam. wide. Cardia 5–7 µm long. Intestine thin-walled, often with a bacterial pouch present in anterior part. Rectum 1.2–1.5 times anal body diam. long, with three rectal glands. Reproductive system didelphic, amphidelphic; ovaries reflexed, not reaching the level of vulva (Fig. 4b, c); anterior ovary on right side and posterior on left side of intestine. Posterior genital branch usually smaller as compared to anterior branch (Fig. 6b, c, d). In several individuals, the posterior branch represented by a post-uterine sac (Fig. 6e). Oocytes arranged in double row in proliferation zone of each ovary. Oviduct a narrow tube leading to a wider ovoid spermatheca (Fig. 4b). The proximal end of spermatheca showing cluster of sperms (sh) developed within the hermaphrodite’s gonad. Uteri containing 2–4 eggs of 50–56 µm x 29–32 µm in dimension occasionally in early stage of segmentation. Each uterus with a large ovoid to kidney-shaped dorsal bi- or trilocular pouch connected to vagina mostly filled with larger sperms (sm) transferred as a result of copulation (Fig. 7). Vagina 17–21 µm, cuticularised, forming an ovijector with thick lumen occupying about 1/4^th^ of corresponding body diam.; epiptygma present (Figs 4c, 7b). Vulval lips slightly protruding. Distance between vulva-anus 0.7–0.9 times tail length. Tail long filiform with a fine pointed terminus.

Male (Figs 2b, f, 5 and Table 1): Similar to female in general appearance but shorter in length and strongly curved in posterior region. Testis single, laterally reflexed, reflexed part 45–52 µm long. Vas deferens a long tube with a tapering ejaculatory duct joining with rectum to form cloaca. Spicules strongly built, heavily cuticularised, strongly arcuate in proximal half, 1.3–1.5 times anal body diam. long with elongated hood-like capitula, a distinct neck and distal part deeply bifurcated with fine extensions and an attenuated ventral arm separated from main body of spicule (Figs 2f, 5a, d, e). Gubernaculum stout, heavily built, 70–82% of spicule length, proximally tapering and curved and distal end with slight protuberances (Figs 2f, 5e). Tail in two parts, an anterior short, conoid part and a posterior long, filamentous part. Genital papillae ten pairs with three precloacal, one adcloacal and six postcloacal pairs. Precloacal pairs GP1 and GP2 closely placed, subventral; GP3 lateroventral. GP4 at level of cloaca. Postcloacal pairs GP5 closely posterior to cloaca; GP6 subventral, more or less one cloacal body diameter posterior to cloaca and nearly at level of phasmids. GP10 subdorsal pair placed slightly posterior to the group of subventrals GP7, GP8 and GP9 (Figs 2f, 5b, c). A membranous rudiment indicating bursa, occasionally confined to posterior most genital papillae including three subventral pairs and one dorsally directed pair (Figs 2f, 5c). Copulatory muscles representing 5-6 pairs of broad bands. Phasmids pore-like, about one anal body diam. posterior to anal opening.

#### Diagnosis

*Acrostichus medius* n. sp. is characterised by female hermaphrodites having a medium-sized body with cuticle bearing faintly striated longitudinal ridges; lateral fields demarcated with two conspicuous ridges; lips amalgamated, labial sensilla small, papilliform; cheilostom with six adradial plates; dorsal tooth moderately-built slightly larger than subventrals; posterior genital branch relatively smaller; dorsal uterine pouch large, multilocular, oval to rounded occasionally filled with sperms and males with robust, heavily cuticularised spicules with hood-like capitula, appearing deeply forked distally with fine extensions and a ventral attenuated arm; gubernaculum 2/3 of spicule length with curved and tapering proximal end and distal end with slight protuberances; bursa almost absent with rudiments confined to posterior four genital pairs.

#### Etymology

The species name ‘*medius*’ is a latin word that indicates the intermediate status of the species showing a blend of characters of *Acrostichus* and *Diplogastrellus*.

#### Distribution

Samples containing *Acrostichus medius* n. sp. were collected from soil rich in organic matter near State Bank of India at Aligarh, Uttar Pradesh, India at geographical coordinates 27°53'35"N, 78°4'27"E.

#### Ecology

**Figure 8.**
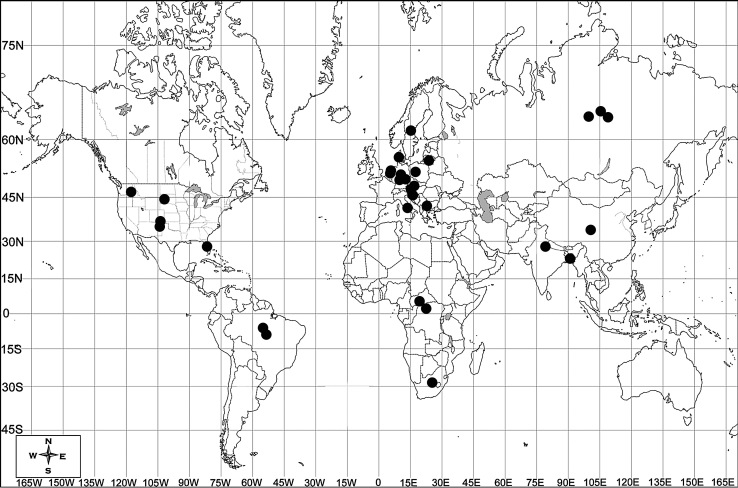
Biogeographical distribution of species of *Acrostichus* Rahm 1928.

*Acrostichus* is a genus with species reported from all the continents except Australia although biogeography of the genus indicates larger distribution in subtropical to temperate regions (Fig. 8). The individuals have been found to inhabit soil, fresh and polluted water and more specifically reported from aquatic mulm or slime flux or sewage; from soil to rotten decaying matter or from moist husk to rotten plants and from dung to frass of beetles. Most species are bacteriophagous but like other diplogastrids, may also feed on protozoa, fungi and nematodes (Bento et al. 2010).

#### Conservation

Due to inhabiting diverse environment types, the species show extensive diversity reflecting phenotypic plasticity. The variations in the shape of buccal cavity which can be shallow and broad, or narrower and deeper, and the variations in size and shape of dorsal tooth are few such examples.

#### Biology

*A. medius* n. sp. shows hermaphrodite females and males in the population. It demonstrates a gradual reduction in posterior genital branch in several specimens up to the extent of a reminiscent post-uterine sac. Such unique feature indicates towards its transitional status in the evolutionary process showing affinities to both the related yet distinct genera *Acrostichus* and *Diplogastrellus*.

#### Taxon discussion

***Relationship with closely related species***

*Acrostichus medius* n. sp. most closely resembles *A. consobrinus* (De Man 1920) Massey 1962 in most morphometric characteristics but differs in having smaller ‘a’ (13.0–19.6 *vs* 20.5–24.3) and greater ‘c' (9.2–15.8 *vs* 7–8) values in females and smaller ‘a’ value (15.2–19.5 *vs* 25.7–29.5) in males; labial sensilla papilliform (*vs* setose); faint (*vs* conspicuous) adradial plates; stoma narrow tubular (*vs* wide tubular); dorsal tooth small (*vs* robust), slightly larger (*vs* markedly larger) than subventrals; spicules with prominently demarcated dorsal and ventral arms having fine distal extensions (*vs* arms not demarcated into arms and genital papillae ten pairs (*vs* nine pairs) in *A. consobrinus*.

*Acrostichus medius* n. sp. also resembles *A. superbus* (Paesler 1946) Massey 1966 in most morphometric characteristics but differs in having smaller ‘a’ value in females (13.0–19.6 *vs* 20.0–24.4) and males (15.2–19.5 *vs* 25.5–30.2); labial sensilla papilliform (*vs* setose); faint (*vs* conspicuous) adradial plates; stoma narrow tubular (*vs* wide tubular); gymnostom (strongly *vs* weakly) cuticularised; subventral metastegostomal teeth relatively larger (*vs* smaller); basal bulb continuous with isthmus (*vs* distinctly demarcated); males with broad, massive (*vs* slender, arcuate) spicules having prominent dorsal and ventral arms (*vs* arms not demarcated) and genital papillae ten pairs (*vs* nine pairs) in *A. superbus apud* Weingärtner 1950 and Gagarin 2002).

The new species comes close to *A. dendrophilus* (Weingärtner 1950) Massey 1966 in most morphometric characteristics but differs in having smaller ‘a’ value (13.0–19.6 *vs* 23.3–30.7) in males; smaller ‘b’ value (5.7–6.3 *vs* 6.8–8.6) in females; greater ‘c’ (3.1–3.6 *vs* 2.0–3.0) and ‘V’ (43.0–46.9 *vs* 33.7–36.7) values; labial sensilla papilliform (*vs* setose); males with broad, massive (*vs* slender, arcuate) spicules; hood-shaped (*vs* rounded) capitula; spicules having prominent dorsal and ventral arms (*vs* arms not demarcated) and genital papillae ten pairs (*vs* eight pairs) in *A. dendrophilus apud* Gagarin 2002.

The new species also resembles *A. lazarevskajae* (Lazarevskaja 1964) Sudhaus and Fürst von Lieven 2003 in most allometric ratios but differs from it in having larger body size (L= 764-867 µm *vs* 365-430 µm in females and 611-715 µm *vs* 290-441 µm in males); greater 'ć' value (9.2-15.8 *vs* 6.6) and large-sized spicules (36-44 µm *vs* 23-25 µm) in males; spicules massive (*vs* slender) with elongated hood-like (*vs* rounded capitula) and distal part deeply bifurcated (*vs* bearing very fine spines at tip) and presence [*vs* absence of an attenuated ventral arm separated from main body of spicules in *A. lazarevskajae apud* Lazarevskaja 1964].

#### Notes

Most of the species of the genus have been described on very flimsy characteristics hence many of them are likely to be synonymous. We tried to have a comparative assessment of the morphological characteristics of most species. Of the total nominal species, few could not be included largely due to insufficient descriptions available.

## Analysis

### Remarks

*A. medius* n. sp. is unique in having a blend of features of *Diplogastrellus* and *Acrostichus*. The species with nearly 1:1 sex ratio in natural population shows hermaphroditism with smaller sperms (sh) stored in spermatheca and the larger ones (sm) filling up the uterine pouch. It may be possible that like *Caenorhabditis elegans*, the males’ sperms besides being larger, have an edge over the hermaphrodites’ sperms in fertilizing the ova. The robust spicules with furcate distal ends, are unique for the genus as well as for Diplogastridae. The well developed and cuticularised ovijector presumably is to complement such spicules during the process of copulation. The gubernaculum, however, shows similarity to those found in several species of *Acrostichus* including *A. superbus*.

## Discussion

### Taxonomic status and affinities of new species

The genus *Acrostichus* is typified by the species *A. toledoi* Rahm 1928. However, the original description of the species lacks some vital information and the illustrations include only the pharyngeal region as well as the male tail region. Massey (1962) considered *Acrostichus toledoi* to be a true representative of the genus with stoma much longer than wide, longitudinal striations prominent, female with a reniform spermatheca and male with massive gubernaculum, almost to the size of spicules and tails of both sexes long and filiform. Andrássy (1984), Andrássy (2005) emphasized on the monovarial condition of the species and, therefore, considered *Acrostichus* to be representative of all monodelphic-prodelphic species. However, the reniform spermatheca of *A. toledoi* seems synonymous to the dorsal uterine pouch which also makes the presence of a single anterior genital branch without a posterior extension, doubtful. Thus *A. toledoi* seems to possess an anterior well developed genital branch and a reduced posterior genital branch occasionally represented by a post-uterine sac. In this perspective, the presence of a relatively well-built upright dorsal tooth, uterine pouch, robust spicules and equally large gubernaculum in *A. toledoi* confirms the status of *Acrostichus* as a senior synonym to *Diplogasteritus* thus supporting the views of Kiontke and Sudhaus (1996). Considering the criterion of single anterior gonad, the species does not seem to enjoy enough affinities with other mono-prodelphic species that belong to genus *Diplogastrellus*. It is also a fact that despite its placement along with other species of *Acrostichus*, the species *A. toledoi* shows some unusual features *viz.*, relatively greater body length (1–1.9 mm *vs* <1mm), relatively elongate (*vs* ovoid) metacorpus, greater ‘V’ value (64 *vs* 35–50) and larger spicules (80 µm *vs* <40 µm in most species of the *Acrostichus*). Nevertheless, in the shape of spicules and gubernaculum, the species resembles *A. superbus* or looks like a close relative of *A. nudicapitatus*, *A. taedus* and *A. gubernatus*. The relative lengths of both genital branches in supposedly amphidelphic genus *Acrostichus*, are likely to be variable hence an unreliable character. The present species *A. medius* n. sp. serves a good example demonstrating a gradual reduction in posterior genital branch (Figs 6b, c, d, e, 7a) in several specimens up to the extent of a reminiscent post-uterine sac. The species also shows variation in the shape of metacorpus that ranged from ovoid to elongate-rectangular type making it a less consistent character for differentiation. It is clearly evident that the representative species of *Acrostichus* clade possess both narrow- and wide tubular but thick-walled stoma with strongly cuticularised dorsal tooth mostly upright and straight or arcuate. Concurrently, the metacorpus in species having narrow tubular stoma is elongate and rectangular type while the species with broader stoma possess a swollen and ovoid metacorpus. Undoubtedly, the uterine pouch is a diagnostic character of *Acrostichus* as also of its junior synonym *Diplogasteritus*. Another reliable feature of the former is the presence of very closely placed precloacals, GP1 and GP2. Thus the amended diagnosis of the genus is as follows:

***Amended diagnosis of genus***

Genus *Acrostichus* can be characterized by the presence of transverse cuticular striations, usually prominent longitudinal ridges; narrowed to truncate lip region; stoma longer than wide consisting of a cuticularized cheilostom with six adradial plates, metastegostom anisotropic, armed with thorn or dagger-like, cuticularized, dorsal tooth and usually smaller subventral teeth; pharynx typically diplogasteroid with metacorpus usually swollen and ovoid, rarely elongate; female gonad primarily amphidelphic with an elongate to bilobed uterine pouch serving as spermatheca, posterior genital branch occasionally reduced; males without bursa, with large cephalated, well-built spicules and usually massive gubernaculum, of variable shape, genital papillae GP1 and GP2 closely placed, tails of both sexes usually filiform.

Most of the species of the genus have been described on sketchy characteristics hence many of them are likely to be synonymous as suggested by Andrássy (1984). The species *A. angustilaimus* (Schuurmans Stekhoven and Teunissen 1938) Massey (1966) has a poor description and illustrations and has also been synonymized with *D. lineatus* by Gagarin (2002). Others like *A. paramicrostoma* (Schuurmans Stekhoven 1943) Sudhaus and Fürst von Lieven 2003, *A. brevicauda* (Schuurmans Stekhoven 1951) Sudhaus and Fürst von Lieven 2003, *A. brevicaudatus* (Schuurmans Stekhoven and Teunissen 1938) Sudhaus and Fürst von Lieven 2003, *A. filicaudatus* (Bütschli 1874) Sudhaus and Fürst von Lieven 2003 and *A. likoi* (Kokordák 1969) Sudhaus and Fürst von Lieven 2003 show insufficient morphological descriptions with the latter two reported on the basis of individuals of one sex only. *A. liratus* (Schneider 1866) Sudhaus and Fürst von Lieven 2003 was described with poor description and no measurements. *A. lazarevskajae* (Lazarevskaja 1964) Sudhaus and Fürst von Lieven 2003, Lazarevskaja 1964, without a proper description, is a new name to *Acrostichus minimus* Lazarevskaja 1964. Further, the species with some anomalies or atypical features were not selected for comparison. *A. paxi* described from five females and five males from decaying wood by Paramonov et al. (1952b) shows a gubernaculum equal to spicules but atypical of *Acrostichus* as also considered species *incertae sedis* by Gagarin (2002). *A. pterygatus* (Timm 1961) Massey 1966 though included in the comparison also shows some unusual features like punctations and the dissimilar stomal armature. *Diplogaster minor* Cobb 1893 as synonymised with *A. minutus* (Kreis 1930) Massey 1966 does not seem to fit in because of presence of a single gonad in female (*apud* Cobb 1893) whereas *D. minor apud* Maupas 1900 shows too wide stoma without characteristic armature. Some of the disparities of descriptions include: descriptions of *A. concolor apud* Massey 1962, Massey 1966 with varying number (6 *vs* 8) of male genital papillae and variation in the shape and size of gubernaculum. Likewise *A. taedus* described by Massey (1962), Massey (1966) do not correspond in the number (7 *vs* 9) and configuration of genital papillae. *A. minimus* (Lazarevskaja 1964) Sudhaus and Fürst von Lieven (2003) as described by Gagarin (2002) shows a disparity in the size of gubernaculum as stated in the text with that shown in illustration.

**Figure 9.**
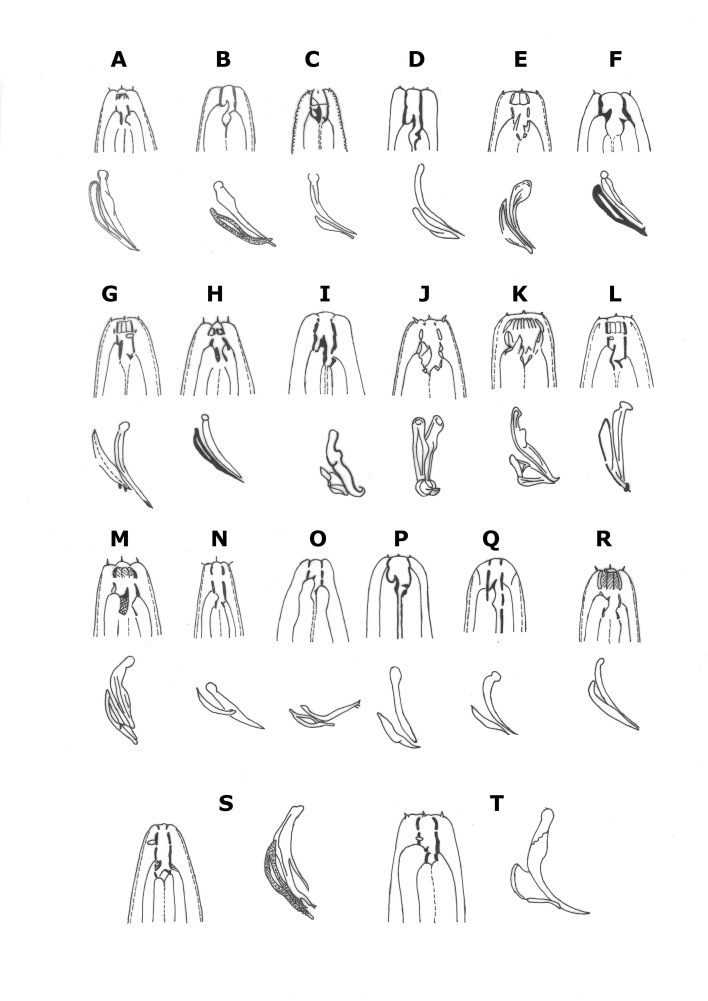
Comparison of stoma, spicule and gubernaculum of selected species of *Acrostichus* Rahm 1928 and *Diplogastrellus cerea* Kiontke and Sudhaus 1996 (modified from the original drawings). (A) *A. аustriасus* (Fuchs 1938) Massey 1962; (B) *A. concolor Massey 1962*; (C) *A. pterygatus* (Timm 1961) Massey 1966; (D) *A. taedus* Massey 1962; (E) *A. rhyncophori Kanzaki et al. 2009*; (F) *A. gubernatus Massey 1974*; (G) *A. nudicapitatus* (Steiner 1914) Massey 1962; (H) *A. occidentalis* (Steiner 1932) Massey 1962; (I) *A. halicti* Giblin and Kaya 1984; (J) *A. megaloptae* Kanzaki et al. 2010b; (K) *A. puri* Kanzaki et al. 2010a; (L) *A. primitivus* (Gagarin 2002) Sudhaus and Fürst von Lieven 2003; (M) *A. superbus* (Paesler 1946) Massey 1966; (N) *A. dendrophilus* (Weingärtner 1955) Massey 1966; (O) *A. lazarevskajae* (Lazarevskaja, 1964) Sudhaus and Fürst von Lieven 2003; (P) *A. stoeckherti* Völk 1950; (Q) *A. lineatus* (Fuchs 1915) Massey 1962; (R) *A. consobrinus* (De Man 1920) Massey 1962; (S) *A. medius* n. sp; (T) *Diplogastrellus cerea* Kiontke and Sudhaus 1996 (figures are schematic and not to scale).

Of the characters taken for cluster analysis (Fig. 1, Table 2) of twenty species of *Acrostichus* Rahm 1928, the stomal characteristics, the presence of uterine pouch, the shape and size of spicules and gubernaculum and the genital papillae as well as the tail shape seem to be important differentiating characters. In the constructed phylogram, *Diplogastrellus cerea* Kiontke and Sudhaus 1996 a species of closely related genus stands out from all species of *Acrostichus* Rahm 1928. *A. medius* n. sp. occupies an intermediate position between the outgroup species and other congeners. The next close relative to *A. medius* n. sp. comes to be *A. consobrinus* (De Man 1920) Massey 1962. However, all the selected species of *Acrostichus* show a more or less orderly grouping. Nevertheless, in the phylogram two large and one small subgroups could be figured out that largely reflect species with closer affinities. The subgroup representing the largest aggregate includes *A. halicti* (Giblin and Kaya 1984) Sudhaus and Fürst von Lieven 2003, *A. puri* Kanzaki et al. 2010a, *A. megaloptaeKanzaki et al. 2010b*, *A. primitivus* (Gagarin 2002) Sudhaus and Fürst von Lieven 2003 and *A. pterygatus* (Timm 1961) [Bibr B2835263]Massey 1966 the former three species shows closer affinity. Another group comprises of *A. dendrophilus* (Weingärtner 1950) Massey 1966, *A. stoeckherti* (Weingärtner 1950) Massey 1966, *A. miminus* and *A. lazarevskajae* with the former three species having more relatedness. *A. taedus* Massey 1962 enjoys affinity with both subgroups. Rest of the species *A. concolor* Massey 1962, *A. rhyncophori* Kanzaki et al. 2009, *A. gubernatusMassey 1974, A. nudicapitatus* (Steiner 1914) Massey 1962, *A. occidentalis* (Steiner 1932) Massey 1962 and *A. lineatus* (Fuchs 1915) Massey 1962 show a staircase arrangement with more or less an orderly grouping except *A. аustriасus* (Fuchs 1938) Massey 1962 and *A. superbus* (Paesler 1946) Massey 1966 which show closer affinities. The clustering groups indicate similarity largely in having slightly to moderately arcuate, simple spicules, trough-shaped, robust gubernaculum of 75-100% of spicule length (Fig. 9). The species *A. rhyncophori* Kanzaki et al. 2009 is unique in the lot with gubernaculum having bifid processes distally. *Titilleus shahinae* Seinhorst et al. 1959b shows striking similarity with the latter and has been raised mainly on the basis of bifid processes of gubernaculum termed as titillae; hence the status of the genus *Titilleus* is doubtful and *T. shahinae* Seinhorst et al. 1959b seems to be a junior synonym of *A. rhyncophori* Kanzaki et al. 2009.

The present species *A. medius* n. sp. seems to serve as a transitional species in the evolutionary process showing affinities to both the supposedly related yet distinct genera *Acrostichus* and *Diplogastrellus*. Besides showing distinctive features of the former *viz.*, thick-walled stoma, a large uterine pouch, primarily amphidelphic female gonad and the closely placed GP1 and GP2, the species shows some distinctive features of *Diplogastrellus* too. There is a tendency of reduction of female posterior genital branch with few individuals possessing only the post-uterine sac. The narrow tubular stoma with a less prominent, triangular dorsal tooth and an elongate to rectangular metacorpus in few specimens also hint towards its affinity with *Diplogastrellus*. Thus the close lineage of the two taxa can further be a matter of investigation as *A. medius* n. sp. seems to be a link between the *Diplogastrellus* and *Acrostichus*. It is also a fact that most species of *Acrostichus* possess prominent cuticular ridges which are often weak or faint in species of *Diplogastrellus*. *A. rhynchophori* and *A. medius* n. sp. form an exception where in addition to faint longitudinal ridges, two prominent ridges are found in the lateral fields. The lip region does not appear to offer any differentiating feature between the two genera as is seen in *A. rhyncophori* and *D. metamasius* Kanzaki et al. 2008 which show striking similarities in the lip regions (*apud* Kanzaki et al. 2009 : Fig. 14 A–C and *apud* Kanzaki et al. 2008 : Fig. 7 A–C). Likewise, the dorsal tooth in *D. indicus* Khera 1970 and *D. sikorai* Khan et al. 2008 appears slightly different from those (Ahmad et al. 2005) typical of *Diplogastrellus* thus making the feature of stoma and dorsal tooth as weak differentiating characters. Thus the only reliable differentiating features of *Acrostichus* are the prominent gubernaculum, uterine pouch and the relative close position of the genital papillae GP1 and GP2.

## Supplementary Material

XML Treatment for Acrostichus medius

## Figures and Tables

**Figure 2a.**
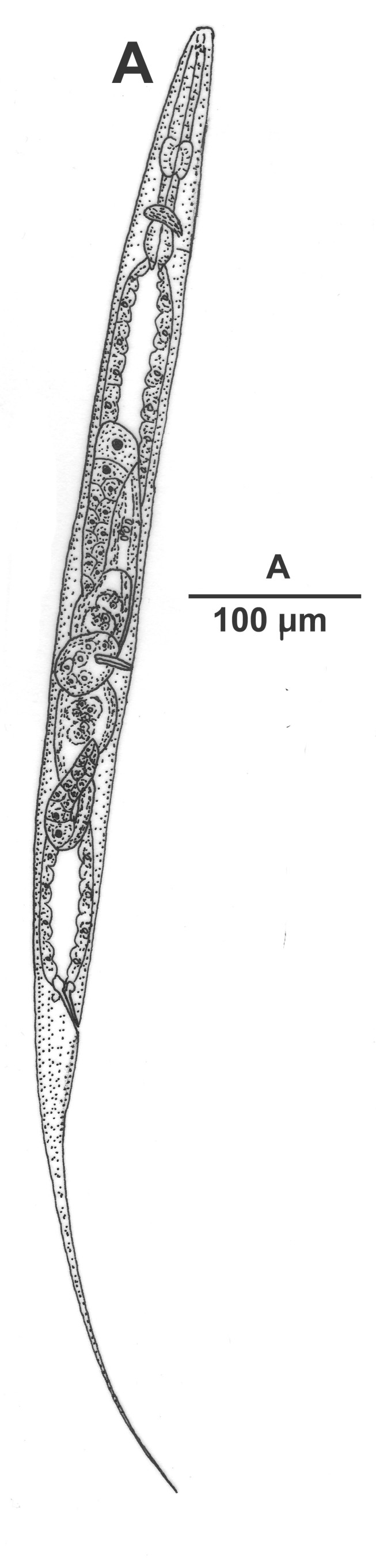
Entire female

**Figure 2b.**
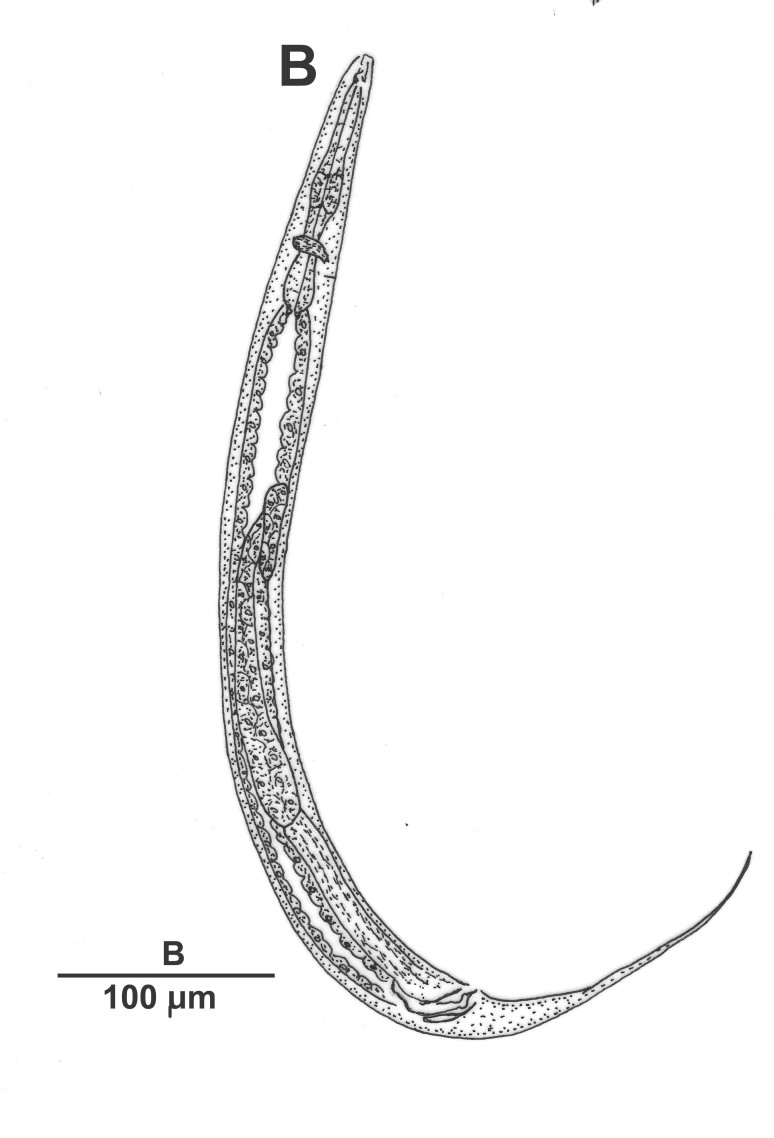
Entire male

**Figure 2c.**
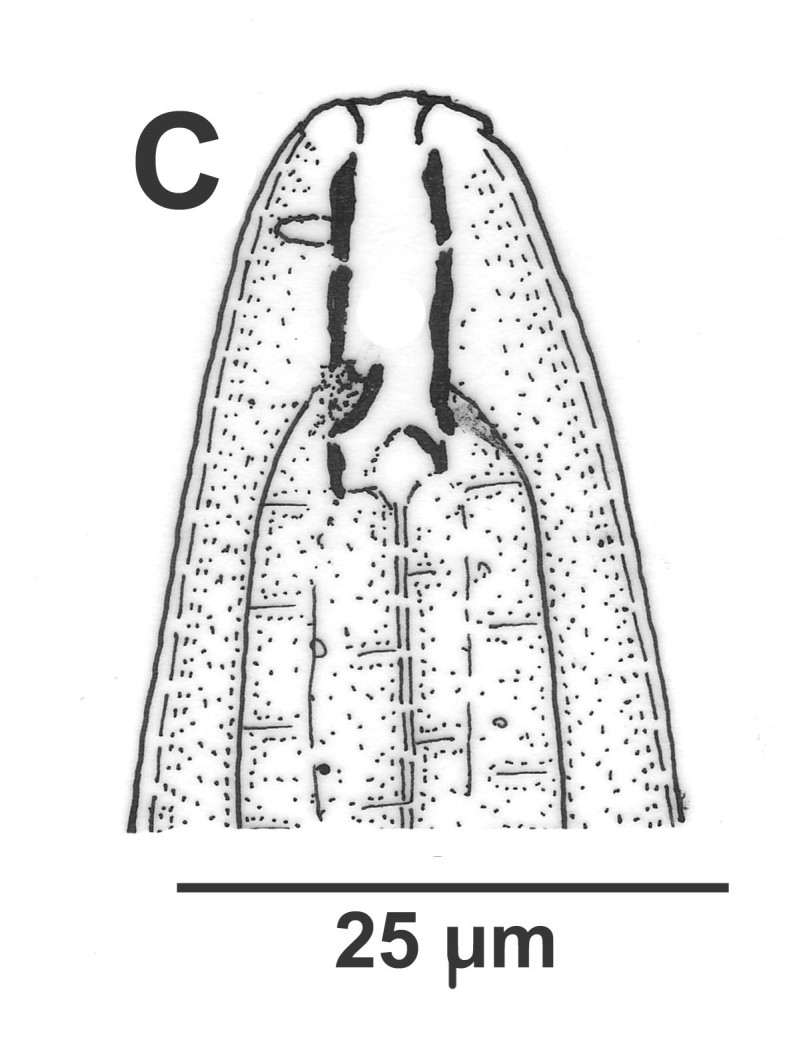
Female anterior end

**Figure 2d.**
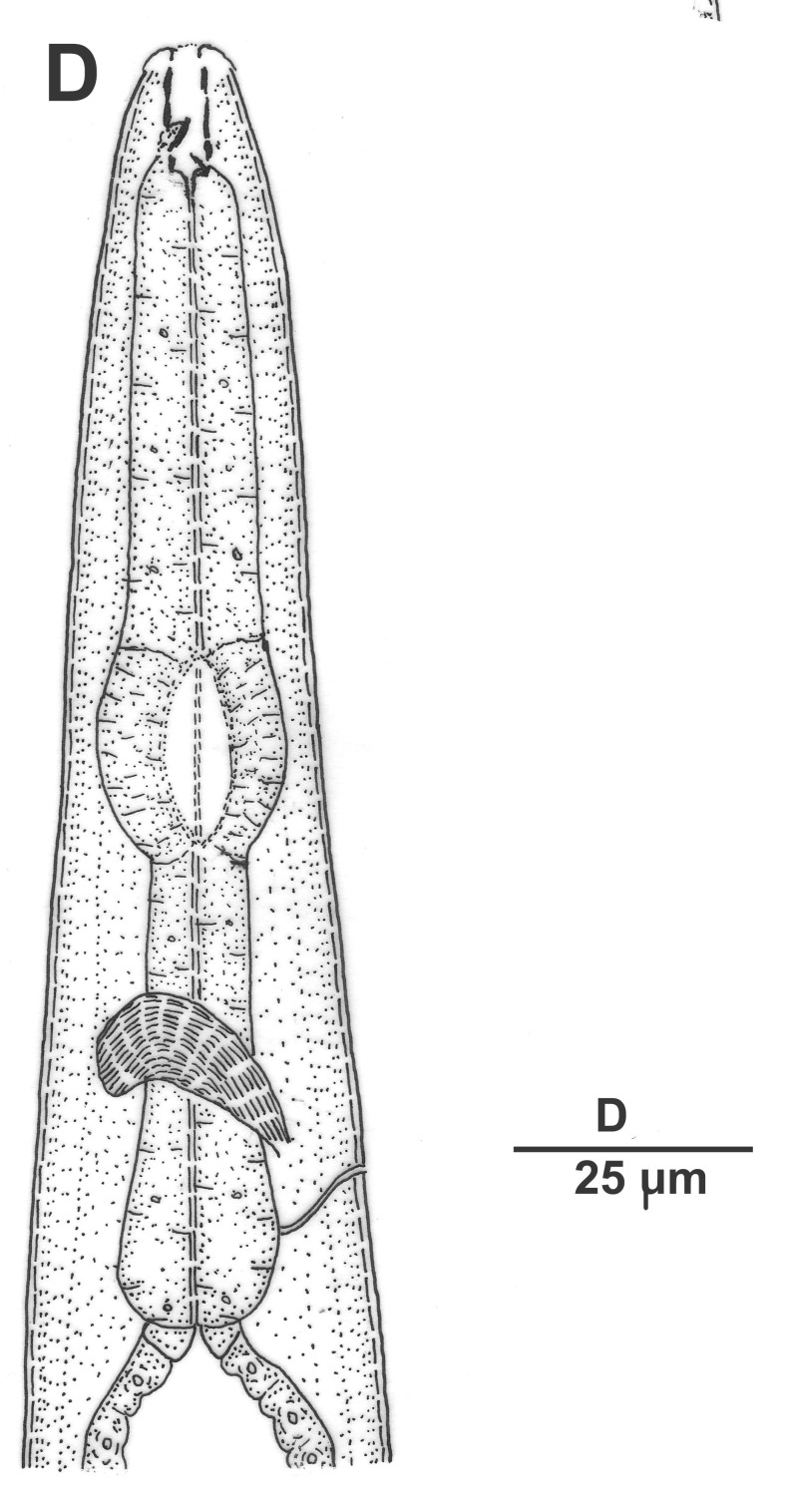
Female pharyngeal region

**Figure 2e.**
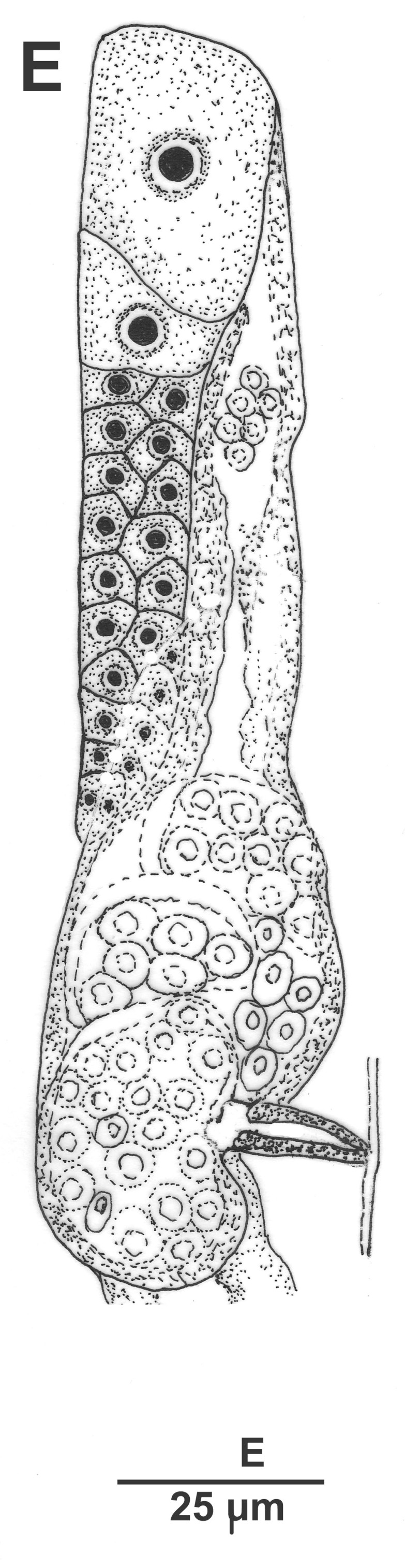
Female anterior genital branch

**Figure 2f.**
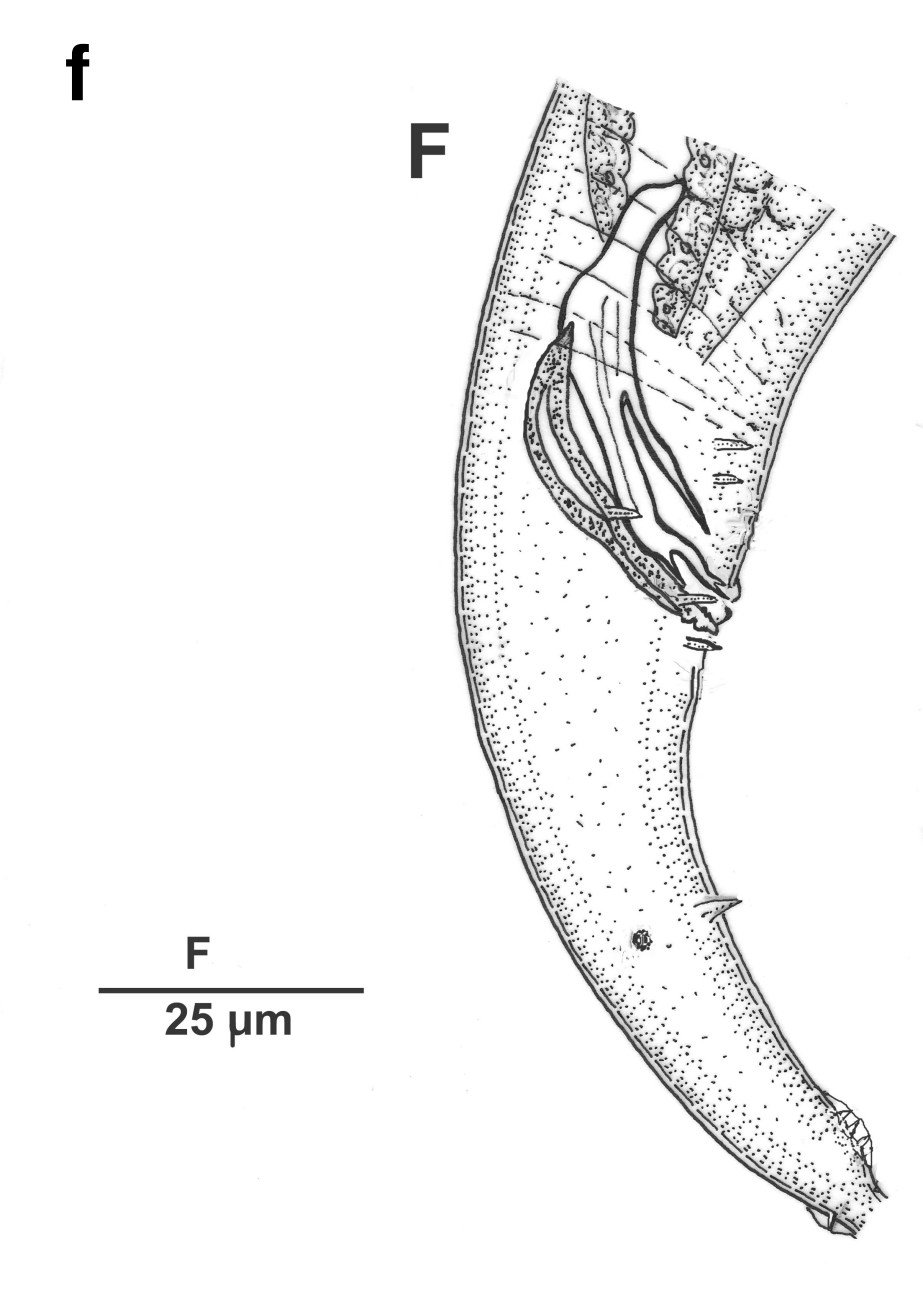
Male cloacal region

**Figure 3a.**
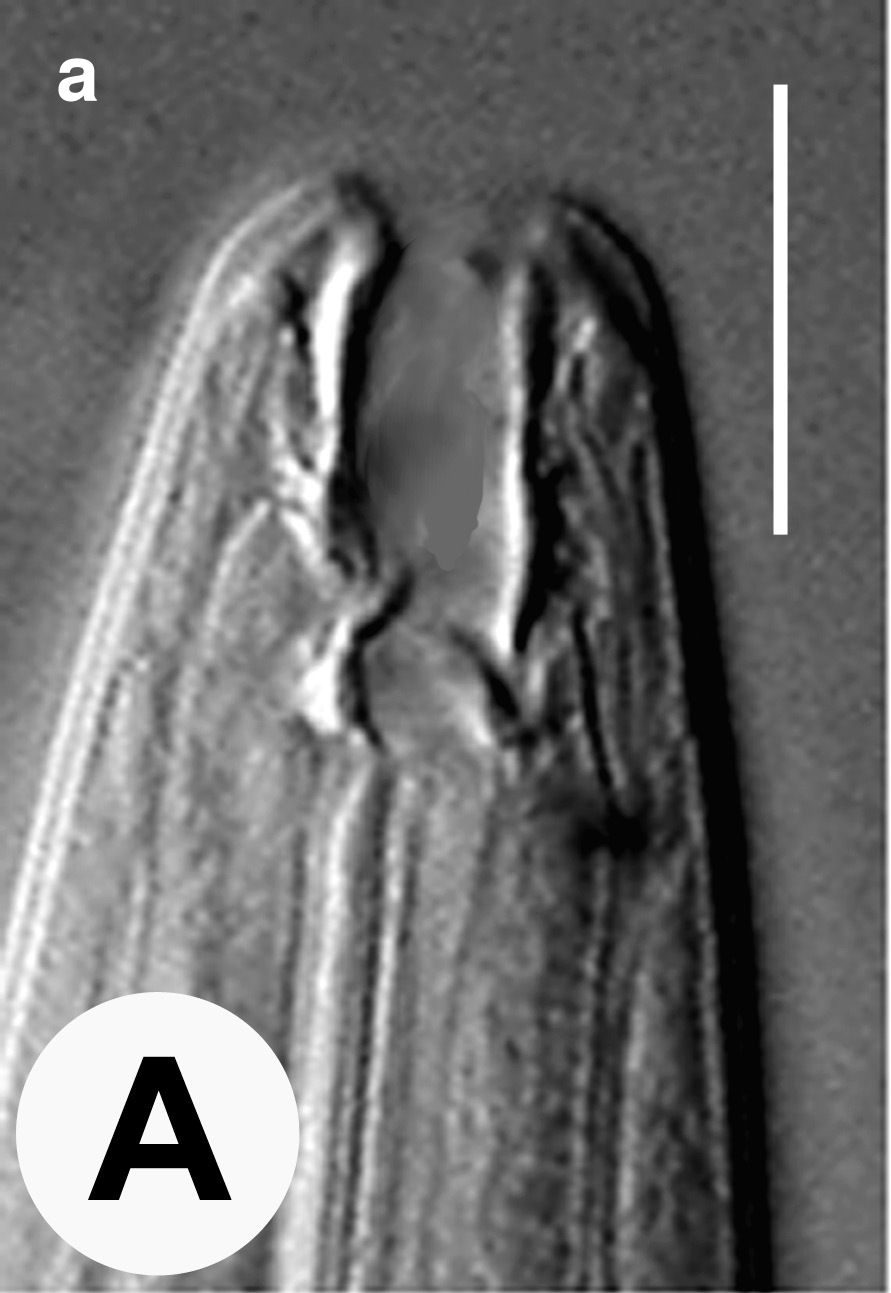
Anterior end (lateral)

**Figure 3b.**
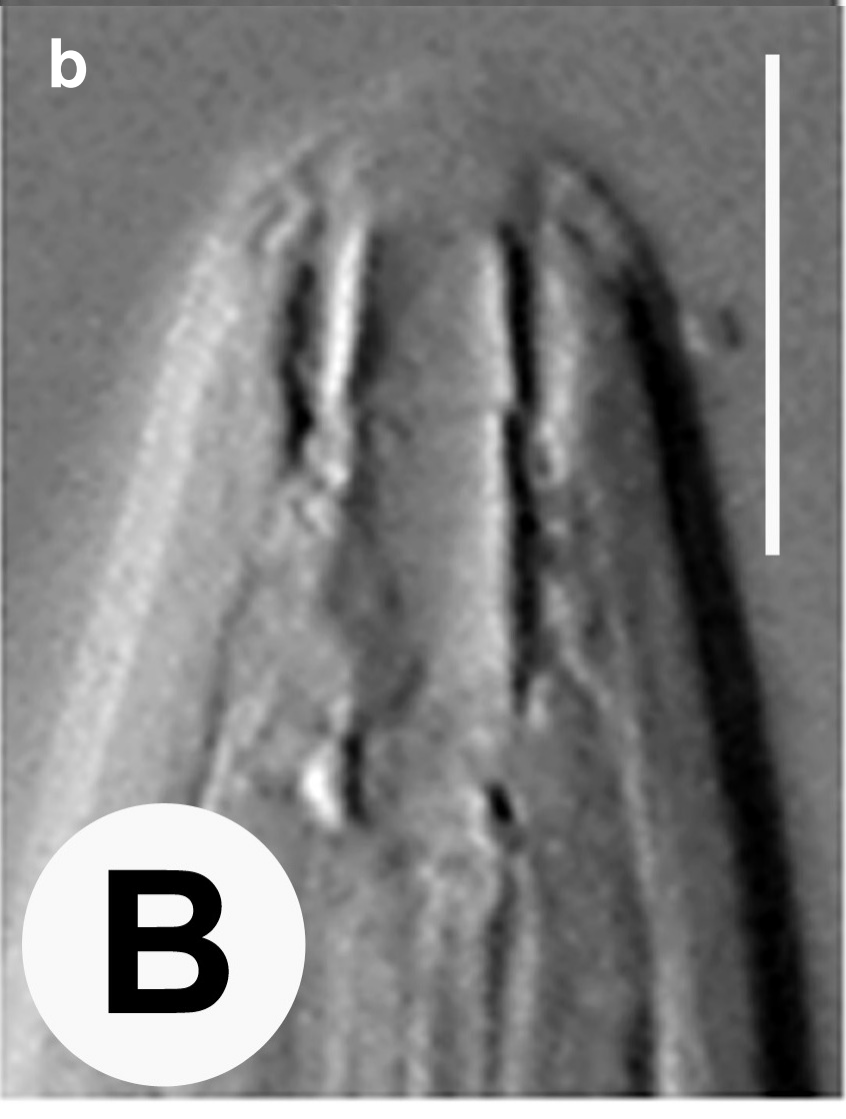
Anterior end (lateral)

**Figure 3c.**
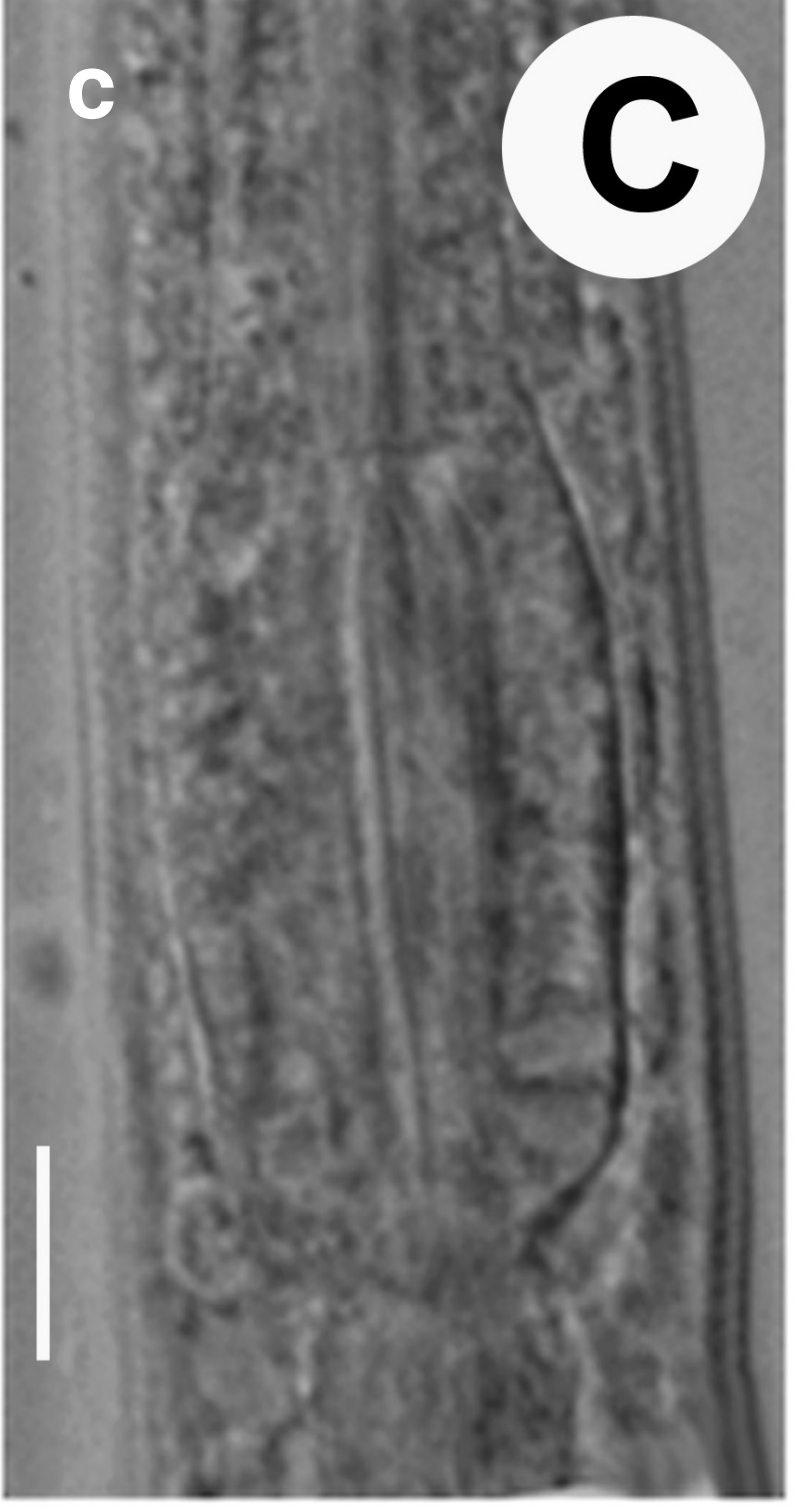
Metacorpal region

**Figure 3d.**
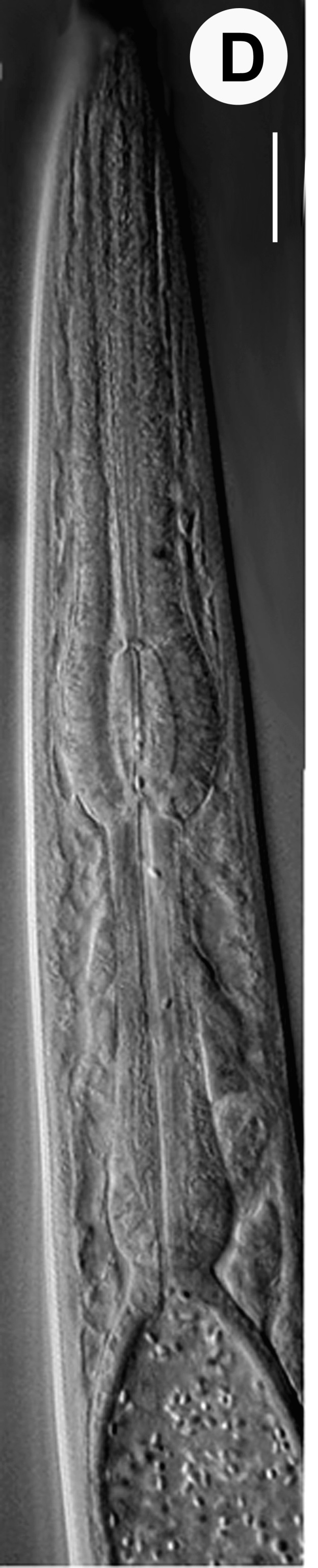
Female pharyngeal region (lateral)

**Figure 3e.**
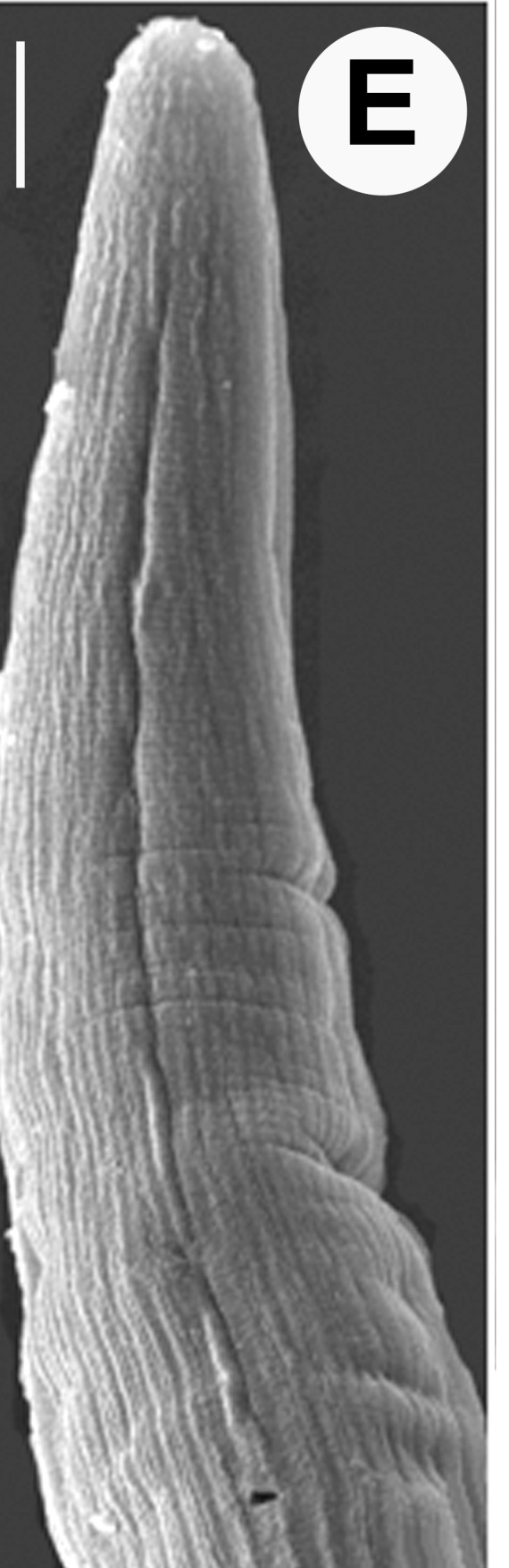
Female pharyngeal region showing secretory-excretory pore (scanning electron microscopy)

**Figure 4a.**
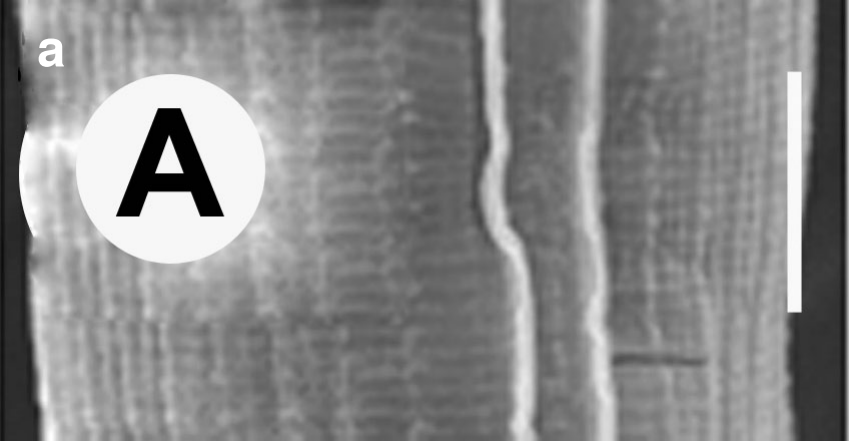
Body region showing lateral fields

**Figure 4b.**
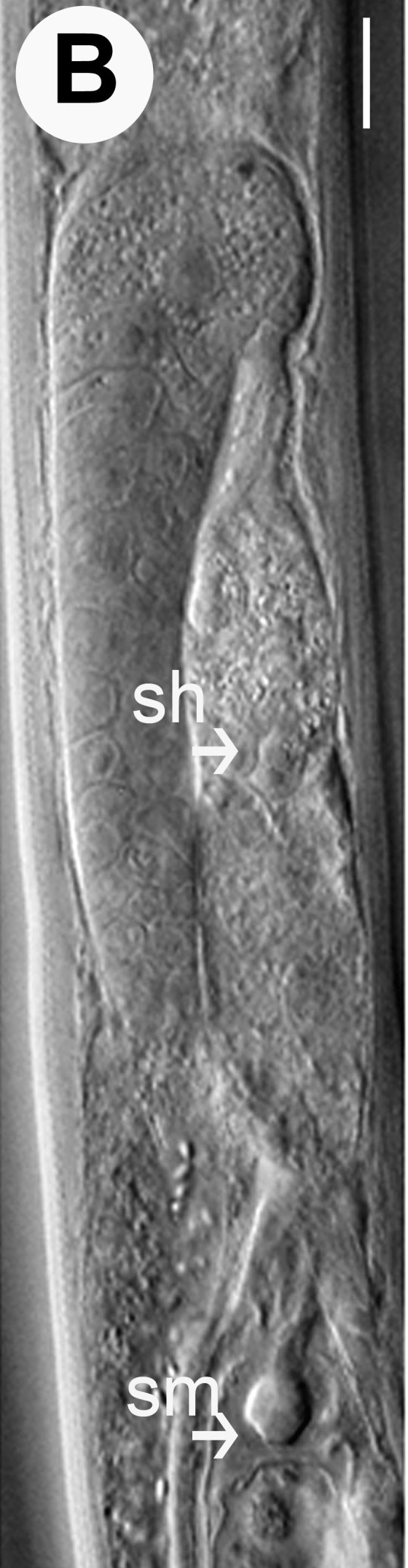
Female anterior genital branch (lateral showing hermaphrodite’s sperms (sh) and male sperms (sm)

**Figure 4c.**
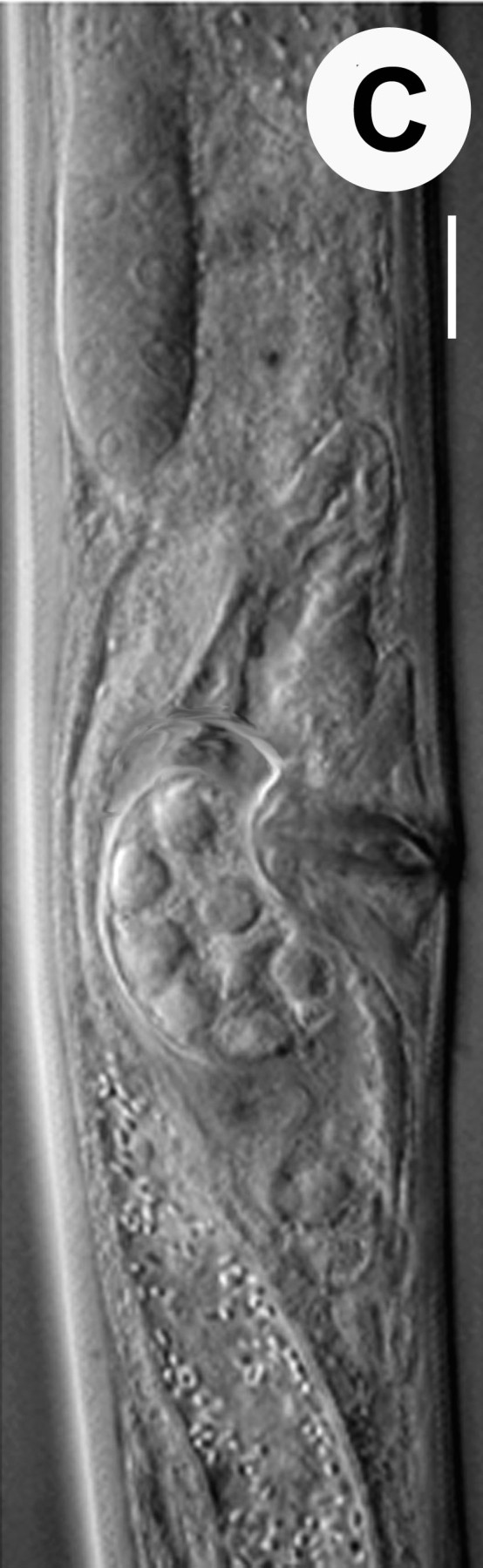
Uterine region (lateral) with ovijector

**Figure 4d.**
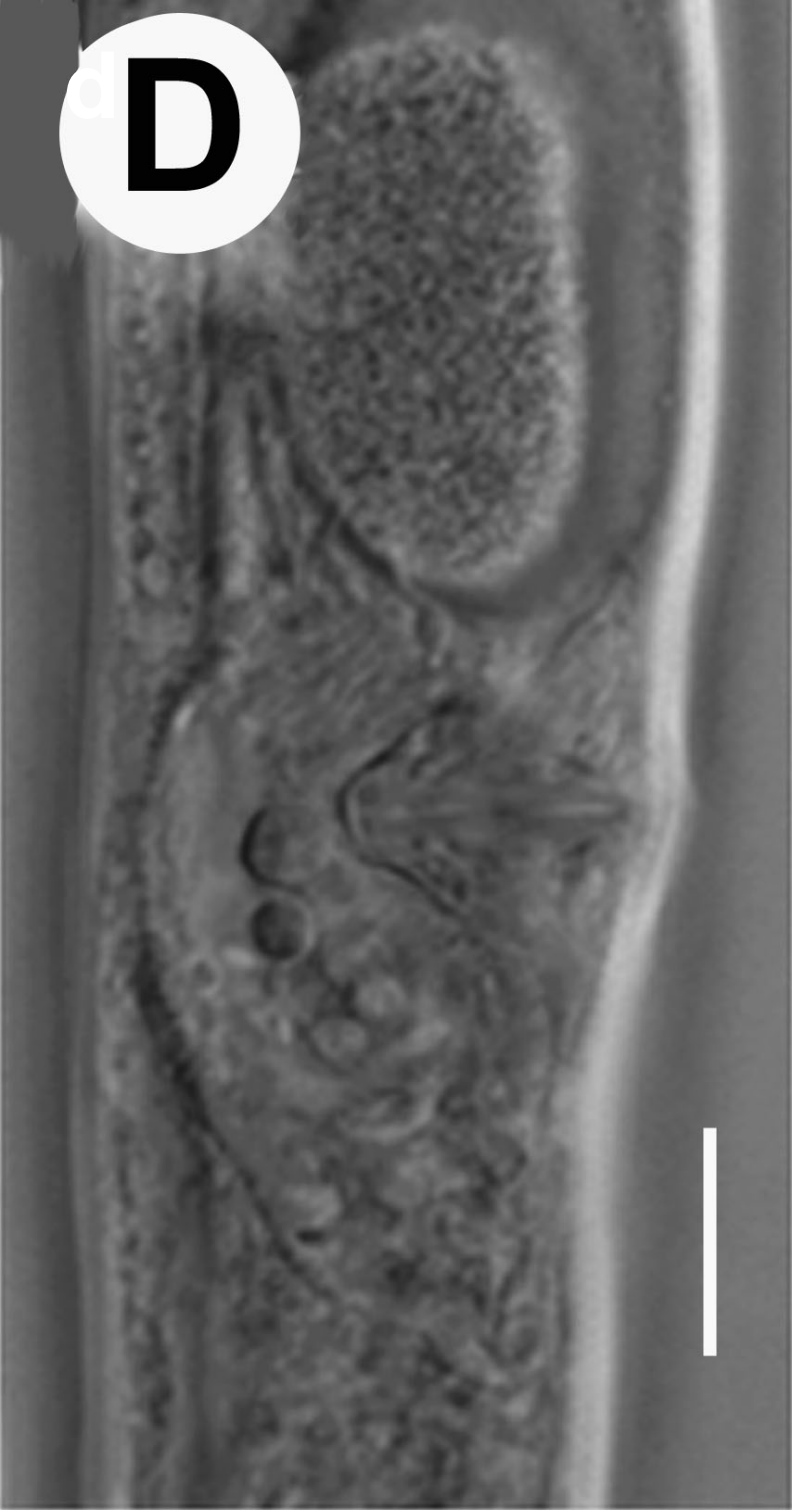
Vulval region with post-uterine sac

**Figure 4e.**
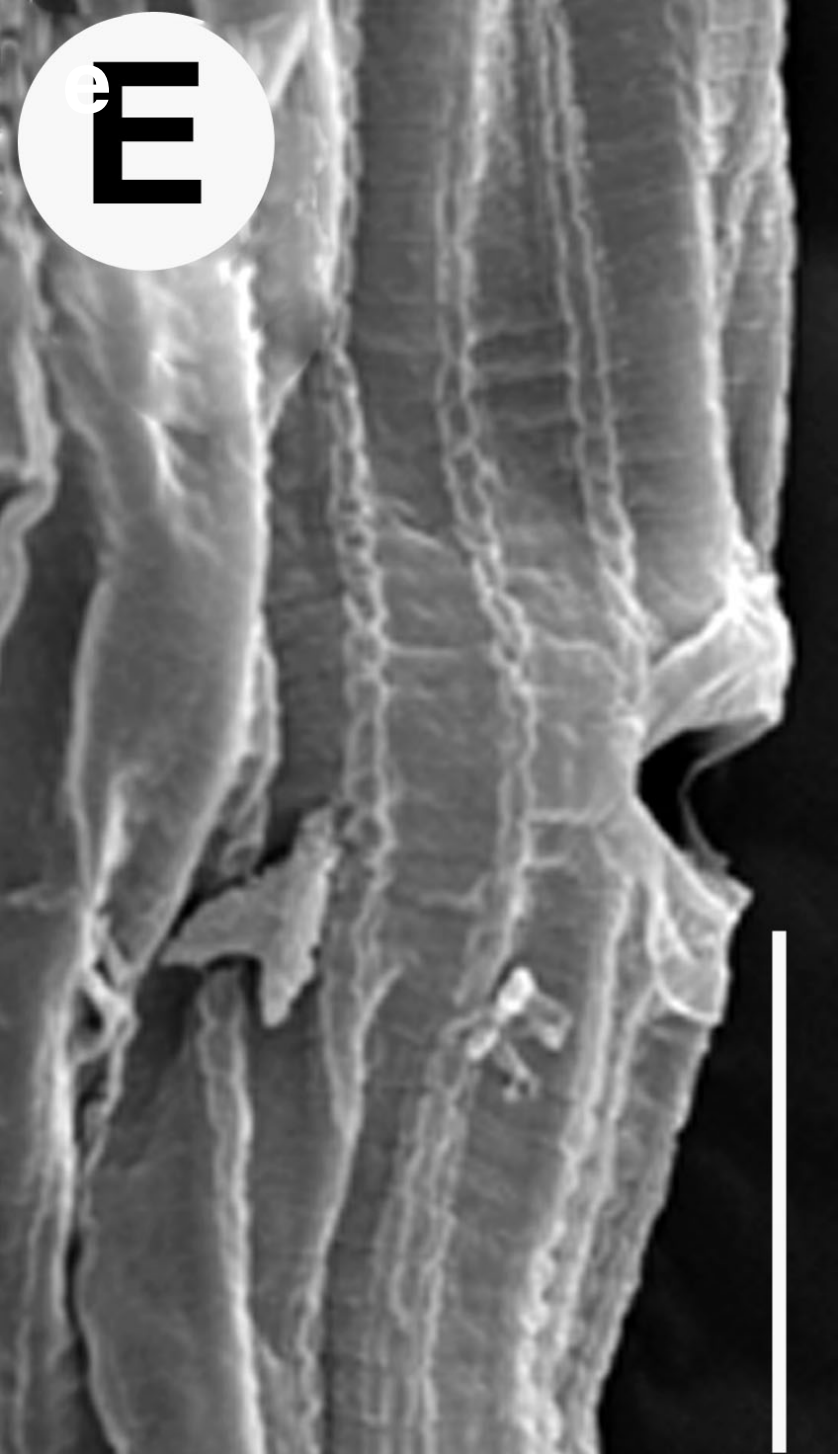
Vulval region (scanning electron microscopy)

**Figure 5a.**
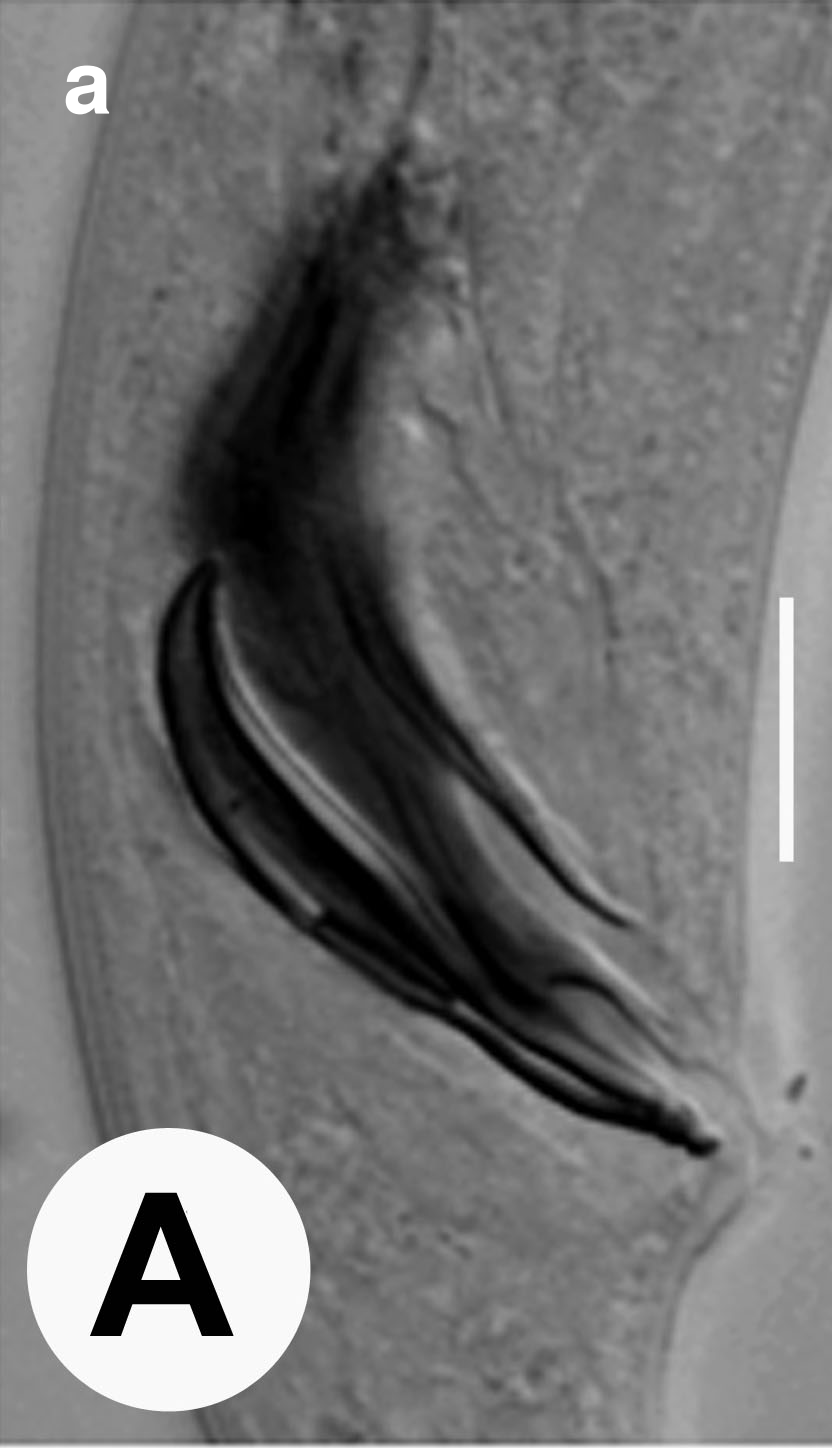
Male cloacal region (lateral)

**Figure 5b.**
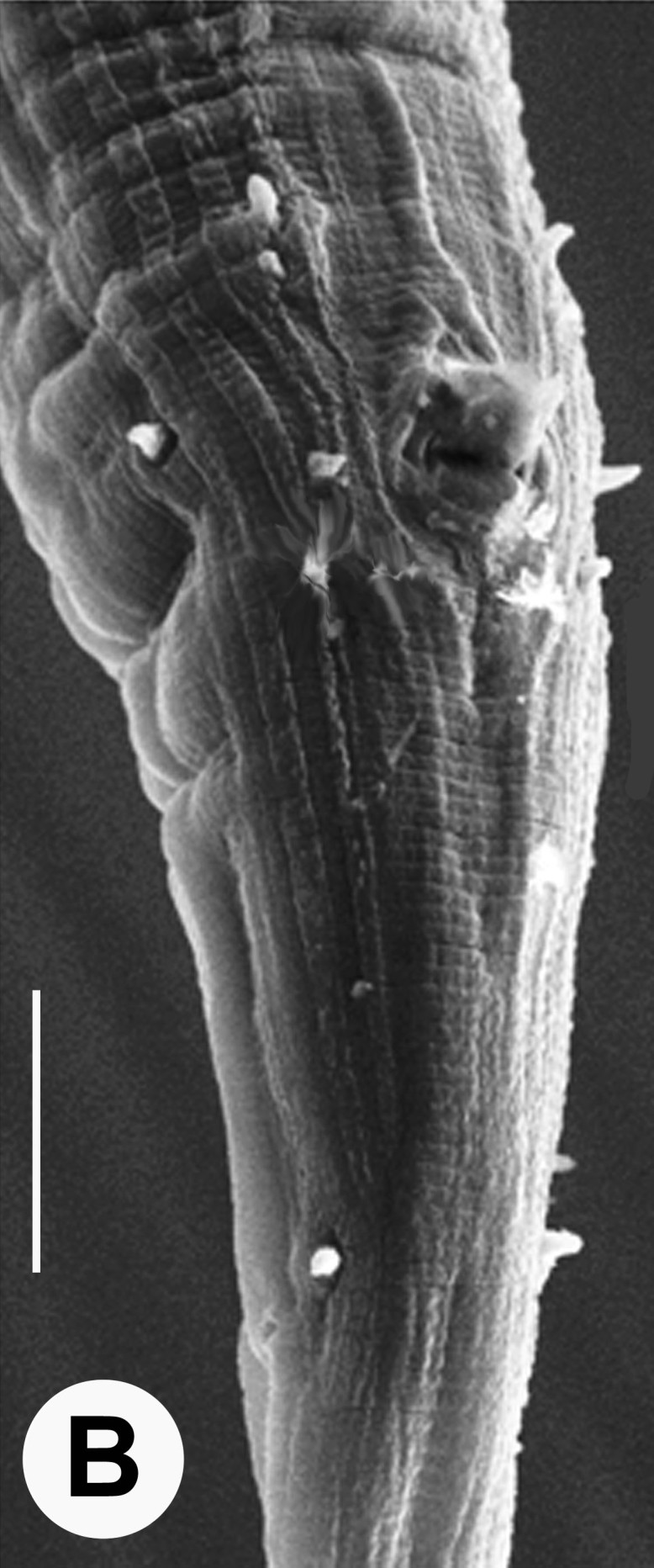
Male cloacal region (ventro-lateral)

**Figure 5c.**
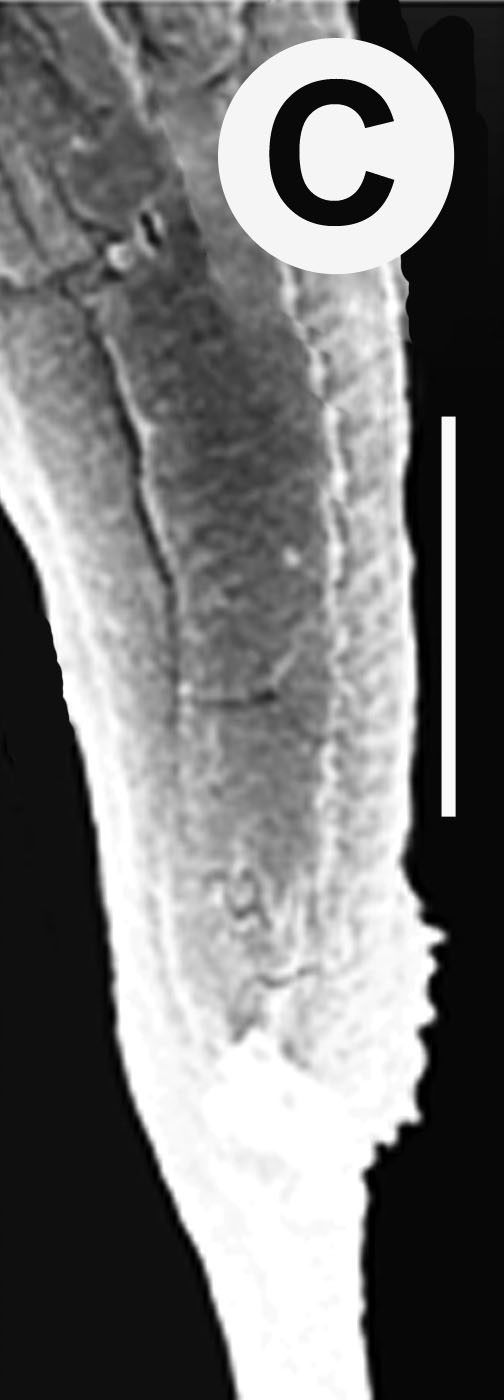
Tail region showing rudimentary bursa

**Figure 5d.**
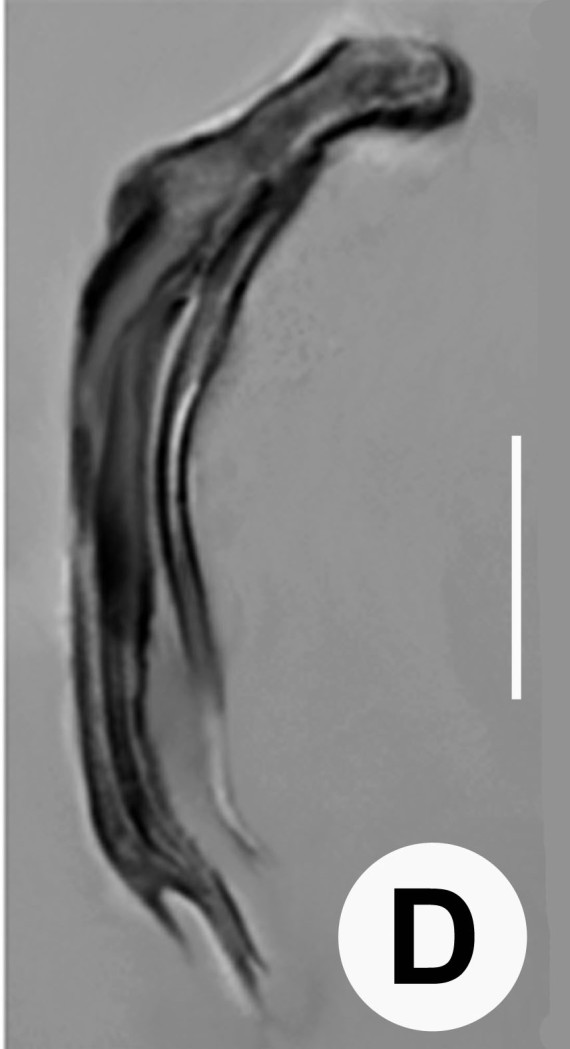
Extracted spicule

**Figure 5e.**
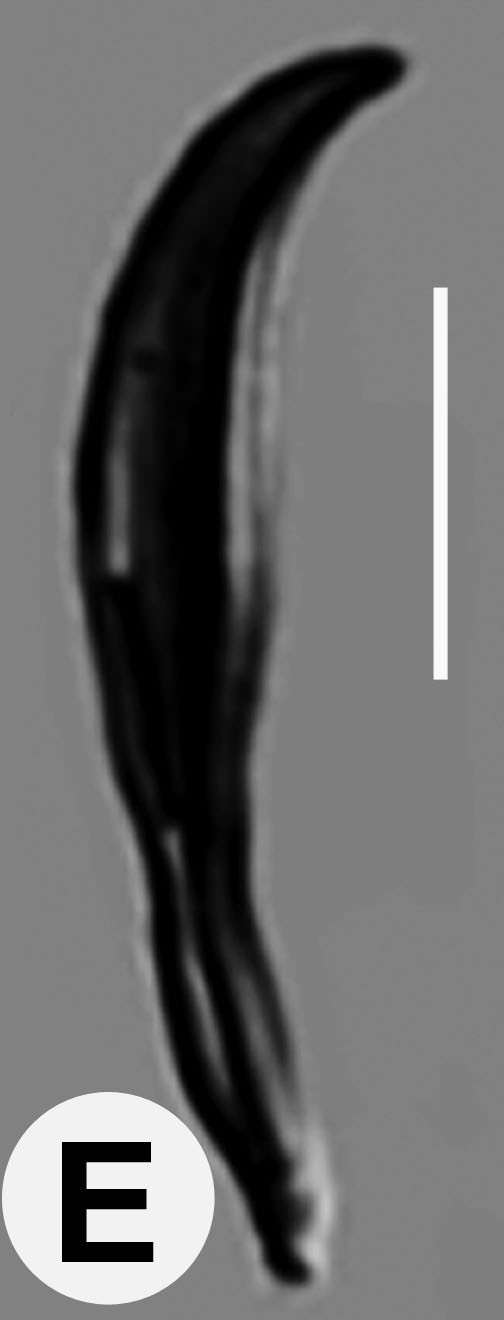
Extracted gubernaculum

**Figure 6a.**
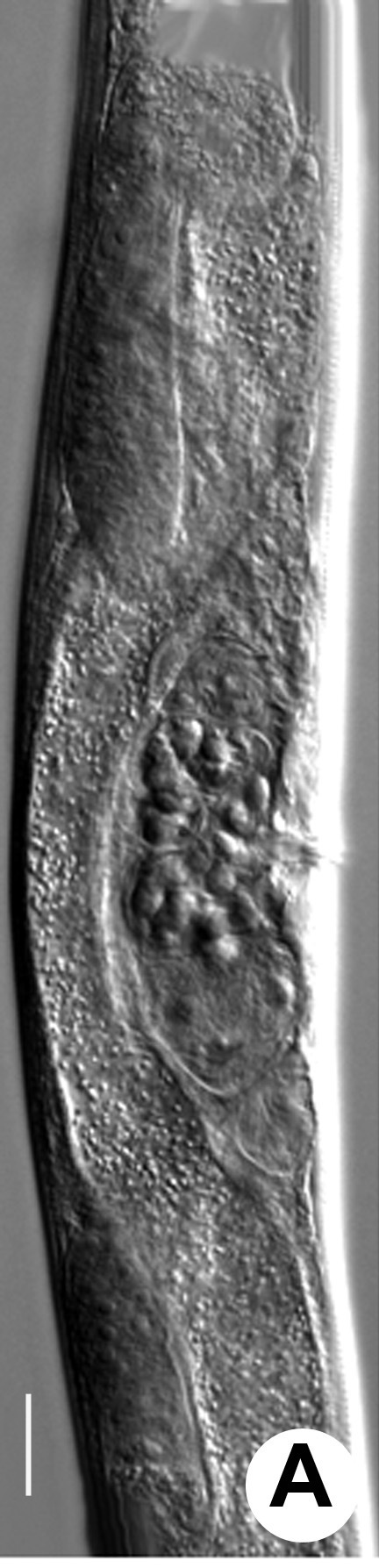
Both genital branches equal

**Figure 6b.**
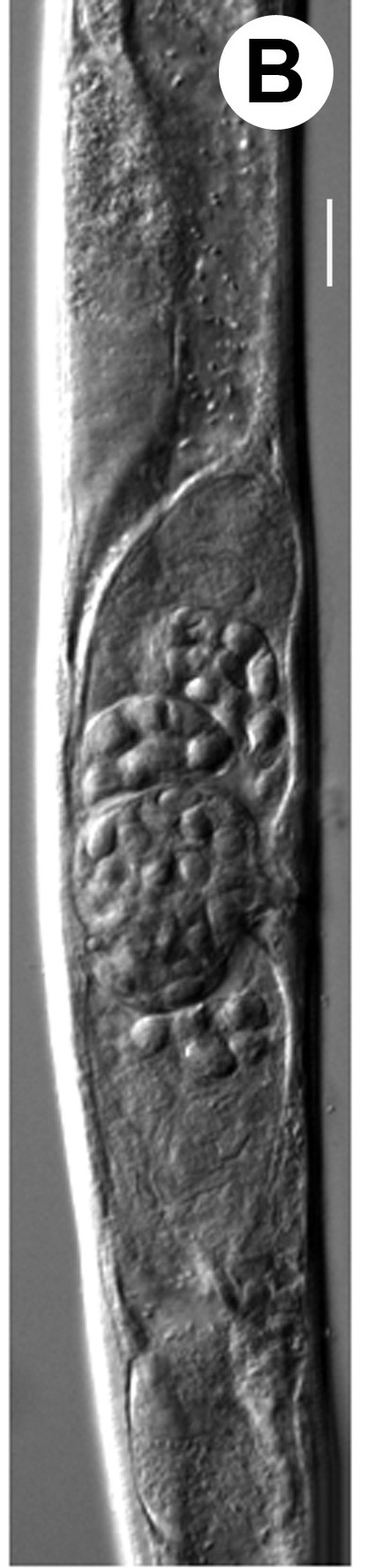
Gradual reduction in posterior genital branch

**Figure 6c.**
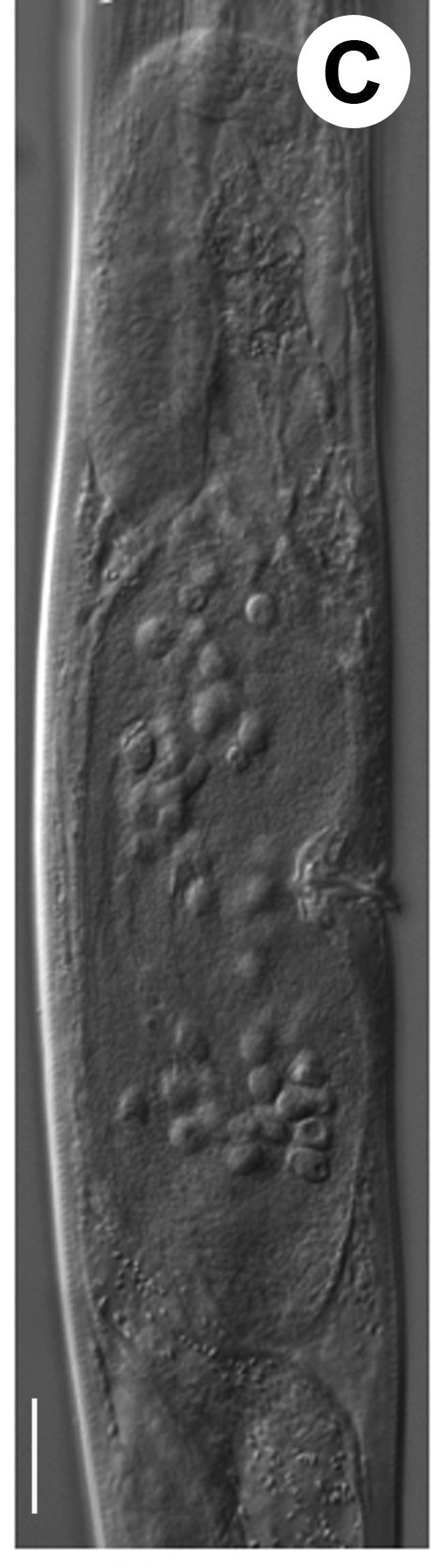
Gradual reduction in posterior genital branch

**Figure 6d.**
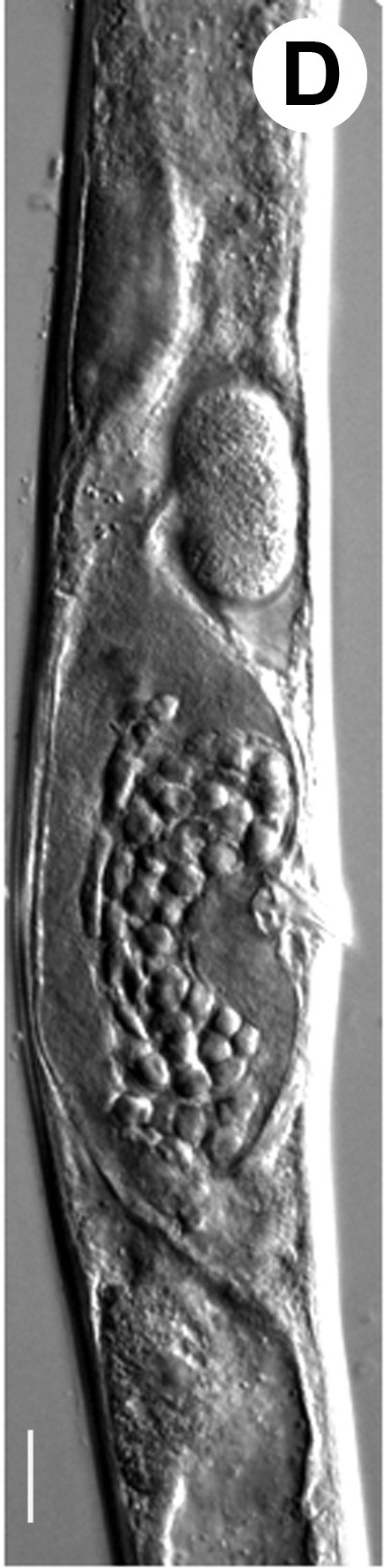
Gradual reduction in posterior genital branch

**Figure 6e.**
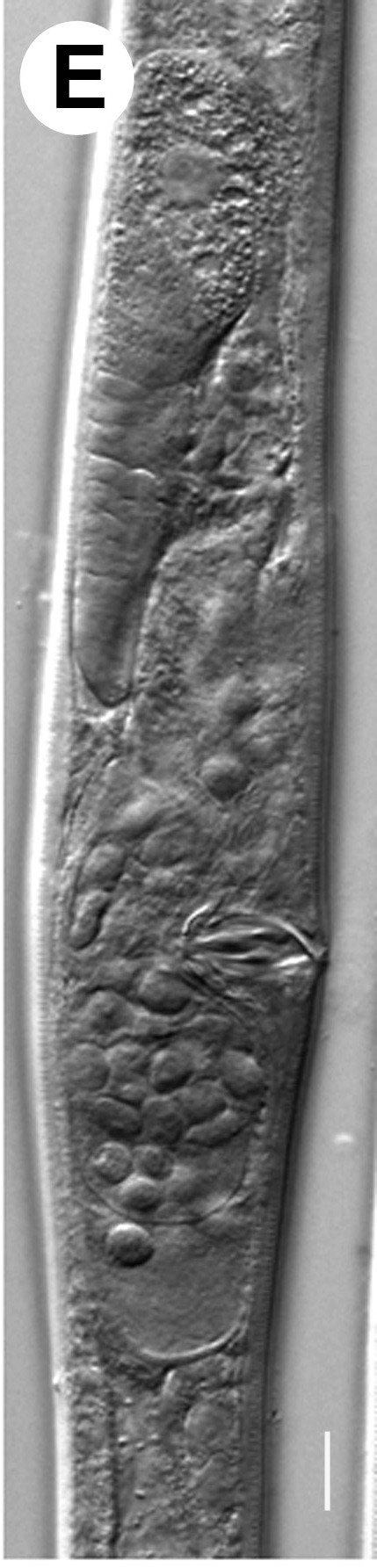
Posterior branch with rudimentary ovary

**Figure 7a.**
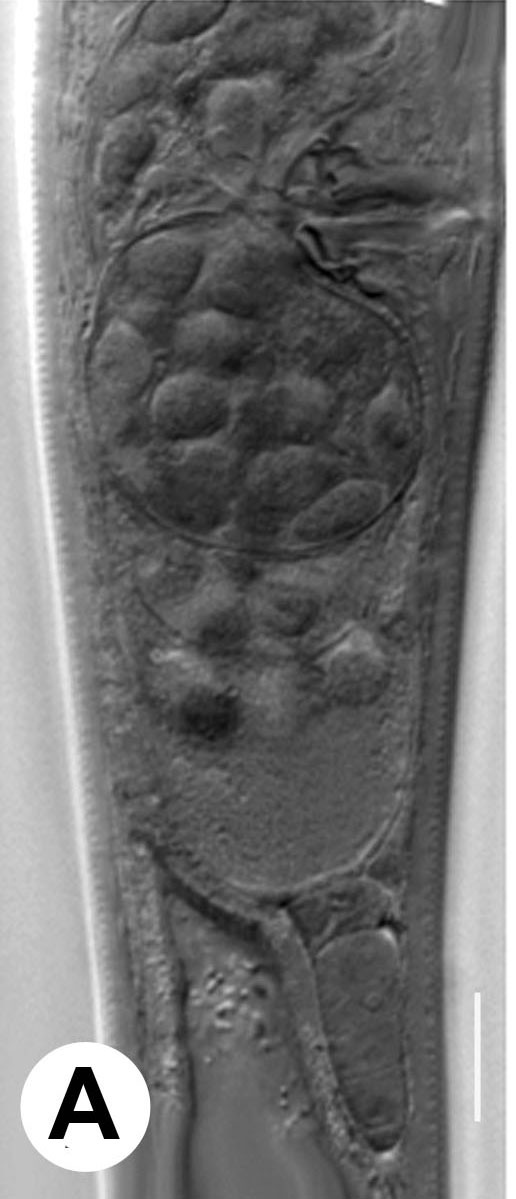
Uterine pouch with attached rudimentary ovary

**Figure 7b.**
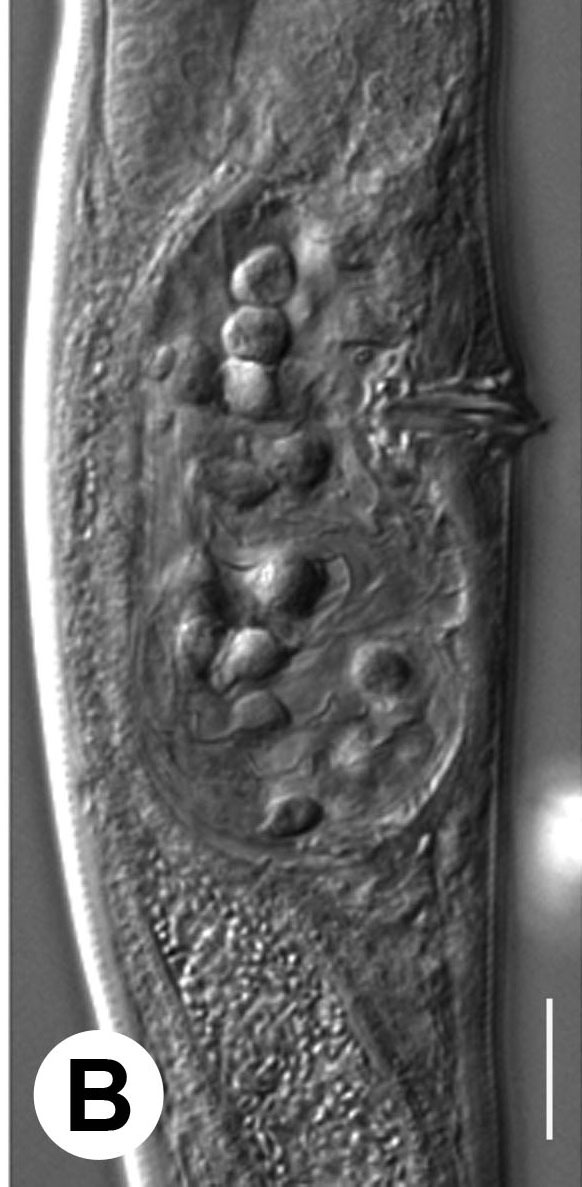
Wide uterine pouch

**Figure 7c.**
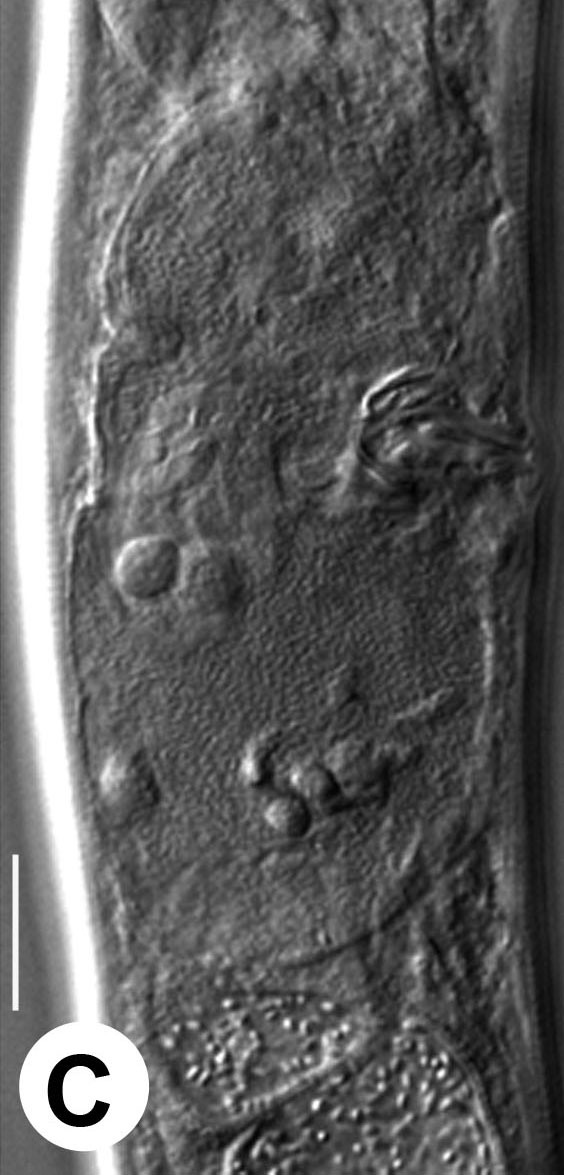
Uterine pouch with several chambers

**Figure 7d.**
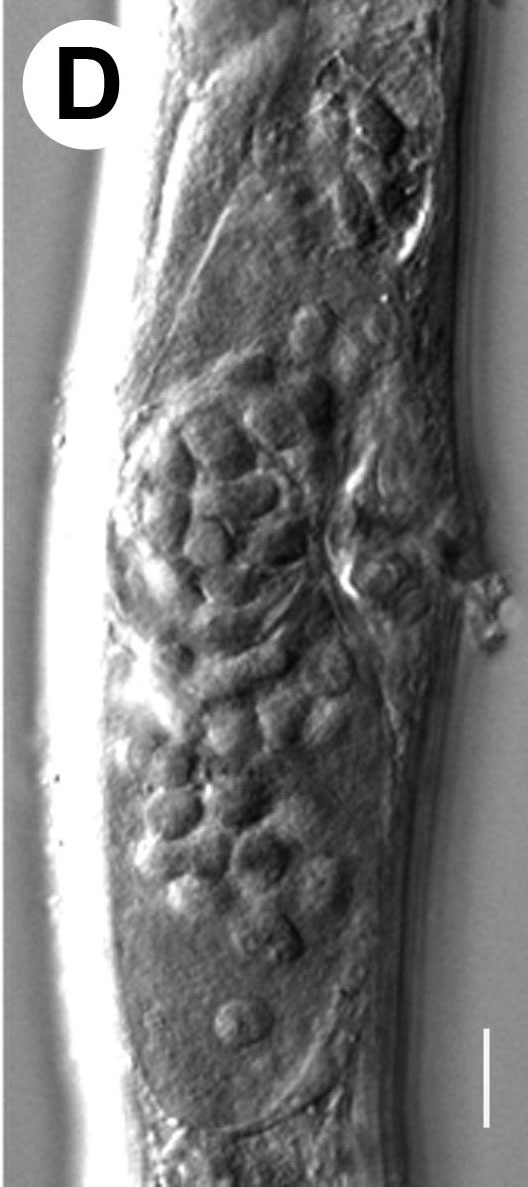
Elongated uterine pouch

**Table 1.** Morphometric characteristics (measurements in µm) of *Acrostichus medius* n. sp.; mean ± standard deviation (range).

**Characters**	***Acrostichus medius* n. sp.**		
	**Holotype male**	**Paratype female** **(n=10)**	**Paratype male** **(n=10)**
Body length	613	821.0 ± 27.5 (764– 867)	659.5 ± 27.6 (611– 715)
Body diameter	33	51.0 ± 7.2 (41– 63)	36.9 ± 2.2 (33– 40)
a	18.6	16.3 ± 2.0 (13.0– 19.6)	18.0 ± 1.1 (15.2– 19.5)
b	5.6	6.0 ± 0.1 (5.7– 6.3)	5.1 ± 0.2 (4.8– 5.5)
c	4.1	3.2 ± 0.1 (3.1– 3.6)	3.7 ± 0.4 (3.1– 4.7)
c’	5.8	11.1 ± 1.7 (9.2– 15.8)	6.4 ± 0.7 (5.6– 7.6)
V/T	49.7	44.7 ± 1.0 (43.0– 46.9)	50.4 ± 3.6 (43.6– 55.5)
G1	-	26.0 ± 2.4 (21.3– 29.0)	-
G2	-	18.9 ± 2.8 (14.2– 22.7)	-
Lip height	3	2.3 ± 0.4 (2– 3)	2.2 ± 0.4 (2– 3)
Lip diameter	7	11.1 ± 0.4 (8– 12)	7.9 ± 0.8 (7– 9)
Stoma length	12	13.6 ± 0.6 (12– 16)	12.2 ± 0.6 (12– 14)
Stoma diameter	3	4.5 ± 0.6 (3– 5)	3.9 ± 0.3 (3.0– 4.5)
Pharynx length	109	136.4 ± 2.1 (133–140)	127.6 ± 5.0 (109– 136)
Nerve ring– ant. end	83	106.4 ± 2.9 (100– 110)	100.0 ± 3.8 (83– 108)
Secretory-excretory pore– ant. end	96	118.4 ± 2.8 (115– 125)	116.3 ± 4.8 (96– 122)
Rectum Length	25	27.5 ± 2.0 (25– 30)	33.5 ± 2.3 (25– 37)
Anal body diameter	26	22.8 ± 2.6 (16– 25)	27.6 ± 1.5 (25– 30)
Tail length	151	249.7 ± 14.9 (228– 274)	178.7 ± 16.0 (151– 195)
Spicule length	39	-	42.0 ± 2.1 (36– 44)
Gubernaculum length	25	-	32.5 ± 1.9 (25– 35)

**Table 2.** Characters and character states for comparison of species of *Acrostichus* Rahm 1928.

**S. No.**	**Character**	**Character state**
1	Female body length	up to 0.7 mm (0), more than 0.7 mm (1)
2	Transverse striations	inconspicuous (0), fine (1), prominent (2)
3	Longitudinal ridges	fine (0), prominent (1)
4	Shape of lip region	truncate (0), rounded (1)
5	Lip region	continuous (0), set off with a depression (1)
6	Labial sensilla	papilliform (0), raised / setose (1)
7	Adradial plates	faint (0), prominent (1)
8	Shape of stoma	narrow tubular (0), wide tubular (1)
9	Stoma length: width	equal (0), two times (1), 2.5-3 times (2)
10	Size of dorsal tooth	small (0), large (1)
11	Shape of dorsal tooth	thorn-shaped (0), claw-shaped (1), weakly triangular (2)
12	Size of subventral teeth	small (0), hardly visible (1)
13	Shape of median bulb	swollen (0), ovoid (1), elongate (2)
14	Valve plates of median bulb	moderately developed (0), strong (1)
15	Shape of glandular part	pyriform (0), rounded (1)
16	Uterine pouch	small chamber (0), spacious complex chamber (1), absent (2)
17	Vulval lips	protruding (0), non protruding (1)
18	Shape of spicules	arcuate (0), straight (1), with angular process (2)
19	Size of spicules	moderately long (0), massive (1)
20	Head of spicule	rounded (0), rectangular/ hood-shaped (1), feebly marked (2)
21	Distal end of spicule	pointed (0), flanged (1), blunt/ rounded (2), hooked (3), divided (4)
22	Shape of gubernaculum	trough shape (0), complex with pieces (1), balloon-like (2)
23	Proximal end of gubernaculum	tapering/ claw-like (0), peg like /or blunt (1), other type (2)
24	Distal end of gubernaculum	pointed with spines/processes (0), pointed without processes (1), blunt without spines (2), complex/hooked (3), with disjointed end (4)
25	Gubernaculum *vs* spicule length	half (0), up to two-third (1), almost equal to spicule (2), about 1/3 (3)
26	GP1 and GP2	closely placed (0), slightly spaced (1), widely spaced (2)
27	Number of precloacal papillae	two (0), three (1)
28	Post cloacal grouped papillae	GP 6-8 (0), GP 5-7 (1)
29	Number of post cloacal papillae	six (0), seven (1)
30	Tail shape	conical spike (0), long filliform (1)

**Table 3.** Data matrix for cluster analysis of the species *Acrostichus* Rahm, 1928. Taxa (T): 1 - *A. austriacus*; 2 - *A. concolor*; 3 - *A. consobrinus*; 4 - *A. dendrophilus*; 5 - *A. gubernatus*; 6 - *A. halicti*; 7 - *A. lazarevskajae*; 8 - *A. lineatus*; 9 - *A. medius* n. sp.; 10 - *A. megaloptae*; 11 - *A. minimus*; 12 - *A. nudicapitatus*; 13 - *A. occidentalis*; 14 - *A. primitivus*; 15 - *A. pterygatus*; 16 - *A. puri*; 17 - *A. rhyncophori*; 18 - *A. stoeckherti*; 19 - *A. superbus*; 20 - *A. taedus*; 21 - *Diplogastrellus cerea*. For characters see Table 2.

**T**	**Characters**																													
	1	2	3	4	5	6	7	8	9	10	11	12	13	14	15	16	17	18	19	20	21	22	23	24	25	26	27	28	29	30
**1**	1	1	0	0	0	1	1	1	2	0	0	1	0	1	0	0	0	0	0	0	0	0	1	0	2	0	1	0	0	0
**2**	0	1	1	0	0	0	0	0	2	1	1	0	0	1	0	1	0	0	0	0	0	0	0	1	2	0	0	0	0	0
**3**	1	1	0	1	0	1	1	1	1	0	0	1	1	0	0	1	0	0	0	1	1	0	0	1	2	0	1	0	0	1
**4**	1	0	1	0	0	0	1	0	1	0	0	1	0	1	0	1	1	0	1	2	1	0	0	0	1	0	0	0	0	1
**5**	0	1	0	1	0	0	1	1	1	1	1	0	0	1	0	1	0	0	0	0	0	0	1	2	2	0	0	2	0	0
**6**	1	0	1	0	0	0	1	1	2	1	1	1	0	1	0	0	0	2	1	3	1	1	2	2	0	0	1	0	0	0
**7**	0	0	1	0	0	0	0	0	2	0	1	0	1	1	0	0	1	0	0	0	4	0	0	2	1	0	0	1	0	0
**8**	1	0	1	0	0	1	1	0	0	0	0	1	1	0	0	0	0	0	0	1	2	0	0	1	0	0	1	1	0	0
**9**	1	1	0	1	0	0	0	0	2	0	2	0	1	1	0	1	1	0	1	1	1	0	1	2	1	0	1	0	0	1
**10**	1	1	1	1	0	0	1	0	2	1	0	0	0	1	0	1	1	1	0	0	1	1	2	3	0	1	1	0	0	0
**11**	0	1	1	0	1	0	0	1	1	0	1	1	1	0	0	0	1	0	0	0	1	0	0	0	1	0	0	0	0	1
**12**	1	2	1	0	1	0	1	1	0	0	1	0	0	1	0	1	0	0	0	0	0	0	0	0	2	0	0	0	0	1
**13**	0	0	1	0	0	1	1	0	0	0	0	0	1	0	0	1	0	0	0	0	0	0	1	0	2	0	0	0	0	0
**14**	0	0	1	0	0	0	1	0	2	1	1	0	0	1	1	0	0	2	0	0	0	1	1	0	1	0	1	0	0	0
**15**	0	2	1	0	0	0	0	0	1	1	1	0	0	1	0	0	1	0	0	0	0	0	1	1	0	0	0	1	0	0
**16**	1	0	1	1	0	0	1	1	0	1	1	1	0	1	0	0	1	2	0	1	2	1	2	2	0	0	1	0	0	0
**17**	0	1	1	1	0	0	1	0	0	1	1	0	0	1	0	1	0	0	1	0	0	1	0	0	2	0	1	0	0	0
**18**	0	0	1	0	0	0	0	0	1	0	0	1	0	1	0	0	1	0	0	0	0	0	0	1	1	1	0	0	1	1
**19**	1	2	1	0	0	1	1	1	1	0	0	0	0	1	0	0	0	0	1	1	0	0	0	0	1	0	1	0	0	1
**20**	0	1	1	0	0	0	0	0	2	1	1	0	0	1	0	0	0	1	1	0	0	0	0	4	2	0	0	0	0	1
**21**	1	1	0	0	0	1	0	0	2	0	2	1	1	0	0	2	0	0	0	0	4	2	2	1	3	2	1	0	0	1
